# Brain age prediction: A comparison between machine learning models using region‐ and voxel‐based morphometric data

**DOI:** 10.1002/hbm.25368

**Published:** 2021-03-19

**Authors:** Lea Baecker, Jessica Dafflon, Pedro F. da Costa, Rafael Garcia‐Dias, Sandra Vieira, Cristina Scarpazza, Vince D. Calhoun, João R. Sato, Andrea Mechelli, Walter H. L. Pinaya

**Affiliations:** ^1^ Department of Psychosis Studies Institute of Psychiatry, Psychology and Neuroscience, King's College London London UK; ^2^ Department of Neuroimaging Institute of Psychiatry, Psychology and Neuroscience, King's College London London UK; ^3^ Department of General Psychology University of Padua Padua Italy; ^4^ Tri‐institutional Center for Translational Research in Neuroimaging and Data Science (TReNDS) Georgia State University Atlanta Georgia USA; ^5^ Georgia Institute of Technology Emory University Georgia USA; ^6^ Center of Mathematics, Computing and Cognition Universidade Federal do ABC São Paulo Brazil; ^7^ Department of Biomedical Engineering School of Biomedical Engineering & Imaging Sciences, King's College London London UK

**Keywords:** biological ageing, healthy ageing, machine learning, regression analysis, support vector machine

## Abstract

Brain morphology varies across the ageing trajectory and the prediction of a person's age using brain features can aid the detection of abnormalities in the ageing process. Existing studies on such “brain age prediction” vary widely in terms of their methods and type of data, so at present the most accurate and generalisable methodological approach is unclear. Therefore, we used the UK Biobank data set (*N* = 10,824, age range 47–73) to compare the performance of the machine learning models support vector regression, relevance vector regression and Gaussian process regression on whole‐brain region‐based or voxel‐based structural magnetic resonance imaging data with or without dimensionality reduction through principal component analysis. Performance was assessed in the validation set through cross‐validation as well as an independent test set. The models achieved mean absolute errors between 3.7 and 4.7 years, with those trained on voxel‐level data with principal component analysis performing best. Overall, we observed little difference in performance between models trained on the same data type, indicating that the type of input data had greater impact on performance than model choice. All code is provided online in the hope that this will aid future research.

## Graphical abstract

We compared the machine learning models support vector regression, relevance vector regression and Gaussian process regression for brain age prediction using different types of morphometric input and sample sizes of more than 10,000 subjects. The mean absolute error across the different models ranged from 3.7 to 4.7 years. The type of data input (region‐ or voxel‐level) had a greater impact on performance than the choice of model.

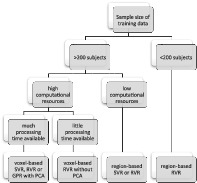

## 1 INTRODUCTION

The world population is ageing rapidly, with one in four people in Europe and North America and one in six people globally predicted to be aged over 65 by 2050 (United Nations, Department of Economic and Social Affairs, 2019). On a societal level, the ageing population is linked to greater socioeconomic costs (United Nations, Department of Economic and Social Affairs, 2019); on an individual level, ageing is associated with a progressive decline in physical and cognitive abilities (Fjell & Walhovd, 2010). It, therefore, is of critical importance to detect age‐related health issues in their early stages to prevent or slow down further deterioration.

Recently, there has been great interest in measuring the ageing process of the brain through brain age prediction using machine learning methods, most commonly based on structural magnetic resonance imaging (MRI). Previous studies have reported very high correlations between brain age predictions and chronological age in healthy people (e.g., *r* > .9, Franke, Ziegler, Klöppel, & Gaser, 2010). In disease, however, the brain‐ageing pattern may deviate from the chronological ageing trajectory. Various psychiatric and neurological diseases appear to have mechanisms that manifest as accelerated ageing in different brain regions, for example, schizophrenia (Koutsouleris et al., 2014; Nenadić, Dietzek, Langbein, Sauer, & Gaser, 2017) and Alzheimer's disease (Franke et al., 2010; Gaser, Franke, Klöppel, Koutsouleris, & Sauer, 2013). These abnormal ageing patterns may be detectable before symptom onset while the individual still appears healthy, and they can serve as a personalised marker of general brain health (Cole & Franke, 2017). Brain age prediction thus has translational potential for early detection of age‐related conditions (Cole & Franke, 2017).

For the successful application of brain age to the clinical context, it is essential to first understand healthy brain ageing and disentangle the effects of different methodological approaches on its prediction. The literature shows great variability in methods, including the choice of analytical models and their parameters, the preprocessing of the neuroimaging data, sample sizes and the selection of input features (e.g., region‐ vs. voxel‐level). While several studies have compared different models on the same data (e.g., Franke et al., 2010; Valizadeh, Hänggi, Mérillat, & Jäncke, 2017), the most suitable methodological approach for brain age prediction is yet to be established.

In this study, we aimed to compare three commonly used machine learning methods to predict brain age: support vector regression (SVR), relevance vector regression (RVR) and Gaussian process regression (GPR). In addition, we aimed to identify the optimal set of processing parameters for each method. Therefore, we assessed the impact of the following methodological choices for models trained on structural MRI data: (a) the use of region‐ or voxel‐based preprocessing of MRI scans, (b) the effect of dimensionality reduction on voxel‐based models, (c) the generalisation of models to an independent data set, and (d) the minimum number of training subjects required for model performance above chance level.

We investigated three main hypotheses. First, based on the previous literature (see overview in Table S1), we expected the models to perform with mean absolute errors (MAE) below 5 years. Second, we expected the models trained on region‐based data to perform better than those trained on voxel‐based data due to the higher dimensionality in the latter, which increases noise, risk of confounding factors and redundancy of data. Third, as RVR is often viewed as state‐of‐the‐art (Franke & Gaser, 2019), we expected this model to achieve the highest level of accuracy for all types of data input, followed by SVR and then GPR. To our knowledge, this is the first study to directly compare these methods on a large data set of more than 10,000 subjects. Our methodologies are introduced in detail and all code is provided online, so that the reader can easily develop and apply the models described here to their own data.

## 2 METHODS

### 2.1 Subjects

The UK Biobank is a population‐based prospective study with over 500,000 participants of middle and old age (https://www.ukbiobank.ac.uk/; Miller et al., 2016; Sudlow et al., 2015). Ethical approval was obtained by UK Biobank from the research ethics committee (REC reference 11/NW/0382). The present study was conducted under project number 40323. We included neuroimaging data from two imaging centres: Site 1, located in the Manchester area (Cheadle), and Site 2, located in Reading. Subjects with a diagnosis of brain‐related disorders were excluded (based on the UK Biobank data code 19 “ICD10”, full list in Table S2). To avoid the confounding effects of scanner differences, we treated the two acquisition sites as separate data sets in further analysis.

We discarded any participants without data available on age, sex, or ethnicity. Participants with non‐white ethnicity were excluded due to the very small sample size, consistent with a previous UK Biobank study took this approach to minimise heterogeneity (Zhao et al., 2018). In order to have large enough samples per age for cross‐validation (Section 2.5.2), age groups with fewer than 99 subjects were excluded, which affected ages younger than 47 or older than 73. In the Site 1 data set, we also excluded subjects to ensure that the male/female proportion would not be statistically different across the different ages (see Figure S1 for details). Based on these criteria, 2,148 out of 12,628 subjects from Site 1 and 77 out of 421 subjects from Site 2 were marked for exclusion. Further subjects were excluded if they did not meet quality criteria for voxel‐based data (Section 2.2.2) or for region‐based data (Section 2.2.4). The demographics of the final included samples are presented in Table 1. The same subjects were used for the region‐ as well as the voxel‐based machine learning analyses.

**TABLE 1:** Demographic information on UK Biobank data set from Sites 1 to 2

	UK Biobank (*N* = 10,824)	
	Site 1 (*N* = 10,480)	Site 2 (*N* = 344)
Age, years		
Mean ± *SD*	61.3 ± 6.9	62.4 ± 6.7
Range	[47, 73]	[47, 73]
Sex, n (%)		
Men	4,734 (45%)	149 (43%)
Women	5,746 (55%)	195 (57%)

### 2.2 MRI acquisition and processing

#### 2.2.1 MRI acquisition

At both sites, structural MRI scans were acquired on a 3T Siemens Skyra scanner with a standard Siemens 32‐channel RF receive head coil. 3D T1‐weighted MRI scans were obtained using a 3D MPRAGE acquisition sequence with the following parameters: inversion time / repetition time = 880/2000 ms, voxel size = 1 mm isotropic, field of view = 208 mm × 256 mm × 256 mm, in‐plane acceleration factor = 2. Further details on the acquisition protocol are available on the UK Biobank website (http://biobank.ctsu.ox.ac.uk/crystal/refer.cgi?id=1977) and in Miller et al. (2016).

#### 2.2.2 Quality control of raw MRI data

Quality control of the raw MRI scans was performed using the freely available machine learning tool MRIQC (Esteban et al., 2017, 2019). MRIQC takes various raw image metrics from an MRI scan, including, for example, the presence of movement, artefacts, and signal‐to‐noise ratio. Subjects were marked for exclusion if they had an MRIQC rating of 0.5 or higher, in line with the default threshold (Esteban et al., 2017). In this way, 1,303 out of 12,628 subjects from Site 1 and 50 out of 421 subjects from Site 2 were marked for exclusion based on the MRIQC score.

#### 2.2.3 Region‐based data preprocessing

Region‐level tissue segmentation and anatomical labelling were performed using the recon‐all pipeline with standard parameters in FreeSurfer (version 6.0, http://surfer.nmr.mgh.harvard.edu/; Fischl et al., 2002). During this pipeline, FreeSurfer automatically removes non‐brain tissue, reconstructs the cortical surface, and segments cortical and subcortical brain regions. The cortical surface of the structural MRI scans was parcellated using the Desikan‐Killiany cortical atlas (Desikan et al., 2006) and segmented into 68 cortical regions (34 per hemisphere). An additional 33 neuroanatomical structures were extracted using the ASEG atlas in FreeSurfer (Desikan et al., 2006; Fischl et al., 2002). Further technical details about the pipeline were described elsewhere (Dale, Fischl, & Sereno, 1999; Fischl et al., 2002). In this study, we normalised the resulting 101 regional volumes (for the complete list, see Table S3) by the total intracranial volume (also computed by FreeSurfer). These normalised regional volumes were used as input data for further analysis.

#### 2.2.4 Quality control of region‐based preprocessed data

Quality control of FreeSurfer‐preprocessed MRI scans was performed using Qoala‐T (Klapwijk, van de Kamp, van der Meulen, Peters, & Wierenga, 2019). Qoala‐T automatically rates the quality of FreeSurfer‐preprocessed scans to detect artefacts or processing errors. We inverted the probability scale of Qoala‐T to match the MRIQC scale (see Section 2.2.2), so that both methods output the probability of a low‐quality image. Subjects were marked for exclusion if they had a Qoala‐T rating of 0.5 or higher in line with the default value (Klapwijk et al., 2019). In this way, 1,626 out of 12,628 subjects from Site 1 and 62 out of 421 subjects from Site 2 were marked for exclusion based on the Qoala‐T score. Overall, subjects were excluded if they were marked as low quality by either Qoala‐T or MRIQC (see Section 2.2.2) or were excluded because of missing data or processing for homogeneity (Section 2.1). Taken together, quality control removed 413 out of 12,628 subjects from Site 1 and 16 out of 421 subjects from Site 2.

#### 2.2.5 Voxel‐based data preprocessing

Voxel‐level preprocessing was performed using the Advanced Normalisations Tools (ANTs, version 2.2.0, http://stnava.github.io/ANTs/; Avants, Tustison, Song, et al., 2011; Avants, Tustison, & Song, 2009). Each MRI scan was first bias field corrected using the N4 method (Tustison et al., 2010) and skull‐stripped using a probabilistic tissue segmentation (via Atropos; Avants, Tustison, Wu, Cook, & Gee, 2011). After the skull stripping, we registered the brain images into a template space called ICBM 2009c nonlinear symmetric (available at http://nist.mni.mcgill.ca/?p=904; Fonov et al., 2011; Fonov, Evans, McKinstry, Almli, & Collins, 2009). The registration was performed using a three‐stage approach that included a rigid body transformation, an affine transformation, and a SyN registration (using the Mattes mutual information) to align each image with the template (Avants, Epstein, Grossman, & Gee, 2008). After this preprocessing, we extracted the voxels inside the template's brain mask and flattened the three‐dimensional volume into a one‐dimensional vector of grey‐scale values that was then used as input to further analysis.

### 2.3 Dimensionality reduction

An important difference between the analysis of region‐ and voxel‐based data is the greater need of addressing the “curse of dimensionality” in the latter, meaning that the number of features (i.e., voxels) is considerably higher than the number of subjects. Methods of dimensionality reduction remove redundant features (e.g., high spatial correlations between voxels) and noise to reduce overfitting of the model to the training data. Principal component analysis (PCA) is a commonly used unsupervised technique for this, in which new features are created by linearly transforming correlated features in the data into a smaller number of uncorrelated features (“principal components”), while retaining most of the variance (Mwangi, Tian, & Soares, 2014).

In the present study, linear incremental PCA (IPCA) from the sklearn library in Python was performed on the ANTs‐preprocessed training data (Ross, Lim, Lin, & Yang, 2008). IPCA is typically used as a replacement for PCA when the data set to be decomposed is too large to fit in memory. IPCA builds a low‐rank approximation for the input data using an amount of memory that is independent of the number of input data samples. It is still dependent on the input data features, but changing the batch size allows for control of memory usage. The only limitation of this method is that the number of components computed must be lower than the batch size. In our case, we were able to compute 150 components without having any numerical issues (when using a batch size of 400 and using 128 GB of RAM). The 150 components explained 71% (±0.002) of the data variance.

### 2.4 Machine learning models

#### 2.4.1 Support vector regression

Support vector machine (Cortes & Vapnik, 1995) is one of the most commonly used supervised machine learning techniques in neuroimaging, especially linear SVR. The main idea behind a linear SVR model is to find a flat hyperplane that deviates from the training data as little as possible, similarly to linear regression. However, in contrast to linear regression where the model aims to minimise the observed training errors, an SVR model calculates the error based only on the data points that fall outside of a so‐called “margin of tolerance” (Smola & Schölkopf, 2003). The margin of tolerance is defined by the hyperparameter epsilon (ε) and represents the deviation from the hyperplane that data are allowed to have. The data points outside of this margin are called support vectors because they determine the position of the hyperplane. Another parameter that influences the performance of SVR is the regularisation hyperparameter *C*. This parameter is used to reduce overfitting by trading off the hyperplane complexity (given by the steepness of the hyperplane) and the obtained training errors. The so‐called “kernel trick” can be used to make nonlinear into linear data by mapping them into higher dimensions through the application of kernel functions (e.g., polynomial, radial basis; Cortes & Vapnik, 1995). However, the present study only used linear kernels because nonlinear methods (a) may require too large sample sizes to generalise well and (b) do not allow for straightforward visualisation making it difficult to determine which regions contributed most to the final model (Rasmussen, Madsen, Lund, & Hansen, 2011).

#### 2.4.2 Relevance vector regression

In contrast to SVR, RVR (Tipping, 2001) uses a general linear model based on Bayesian inference, meaning that its predictions are probabilistic instead of deterministic. The latter is achieved by assuming a prior probability distribution of the weights of the input data as a zero‐mean normal distribution and iteratively adjusting the values of precision in the model using evidence approximation. While training, the weights with weak precision are set to zero, and the basic functions associated with it are pruned. RVR results are generally sparser than SVR, that is, they use fewer support vectors, which contributes to their greater robustness to outliers and higher generalisation (Wang, Fan, Bhatt, & Davatzikos, 2010). Furthermore, since RVR does not require hyperparameter tuning, it avoids the need to run methods like grid search or random search, thus making the RVR training process potentially less computationally expensive (Franke & Gaser, 2019). However, because the learning method is a variation of expectation maximisation, the optimisation is non‐convex, which makes the predictions more prone to local minima errors.

#### 2.4.3 Gaussian process regression

GPR represents a nonparametric Bayesian approach to classical regression in form of a supervised machine learning model (Rasmussen & Williams, 2006; Williams & Rasmussen, 1996). Instead of learning the exact target value of training data, GPR infers a probability distribution of possible values. Performing GPR requires the specification of a prior distribution as a mean and covariance kernel (e.g., linear, nonlinear, radial). It is usually assumed to be a multivariate Gaussian distribution with mean 0. The probabilities of this prior distribution are then adjusted based on the target values in the training data using Bayes' theorem. In the resulting posterior distribution, the information from the prior distribution and the real data are combined into joint probabilities. If the prior distribution is assumed to be Gaussian, the predictive distribution for previously unseen data will also be Gaussian. From this predictive distribution, the prediction for a previously unseen value can be inferred as the mean and the uncertainty of the prediction as its variance. RVR is a sparse version of GPR with a specified covariance kernel.

### 2.5 Model development and comparison

#### 2.5.1 Model training

##### Region‐based models

Region‐based SVR, RVR and GPR models were trained on all subjects from Site 1 that passed the quality checks of raw and segmented data (Sections 2.2.2 and 2.2.4).

For the trials using FreeSurfer data, each region was first normalised by total intracranial volume and then separately scaled using statistics from the training set. Using the robust scaler approach in the Python package scikit‐learn (Pedregosa et al., 2011), we removed the median and scaled the values of the regional data to the interquartile range to increase robustness to outliers. These scaled values served as input to the model training.

Linear SVR was implemented using “LinearSVR” in the scikit‐learn package with an epsilon‐insensitive loss function. SVR models require tuning of hyperparameter C (see Section 2.4.1). Within each iteration of the cross‐validation (CV, see Section 2.5.2), a fivefold nested CV (stratified by age) was implemented to conduct a systematic hyperparameter search for C using the sklearn grid search method over the search space 2^−7^, 2^−5^, 2^−3^, 2^−1^, 1, 2, 2^3^, 2^5^ and 2^7^. The scoring parameter was specified as negative MAE. The model with the best hyperparameter value (measured by the MAE) was then retrained on the whole training set from that CV iteration before it was applied to the test set. All other parameters of linear SVR were used with their default values, for example, an epsilon value of 0, a tolerance for stopping criterion of 1e‐4, and a maximum of 1,000 iterations.

RVR models were trained using the Python library sklearn‐rvm, which implements expectation‐maximisation RVR. The models were specified to use a linear kernel and a threshold for alpha selection criterion for the number of relevance vectors of 1e9. The default values were used for all other parameters, such as a tolerance for stopping criterion of 1e5, an unfixed beta, no prespecified initial value for alpha, no bias added to the decision function, and a maximum number of 5,000 iterations.

GPR was implemented using “gaussian_process” in the scikit‐learn package with a dot‐product kernel to specify the covariance function. The remaining parameters of the function were left at default, for example, an alpha value of 1e‐10 and an optimiser “fmin_l_bfgs_b” with only one run, which determines the optimiser for the hyperparameter theta.

##### Voxel‐based models

Voxel‐based SVR and RVR models without PCA as well as the voxel‐based SVR, RVR and GPR models with PCA were trained on all subjects from Site 1 that passed the quality checks of raw and segmented data (Sections 2.2.2 and 2.2.4), so the same subjects were used as input to the region‐ and voxel‐based models.

The kernels and parameters for the voxel‐based models without PCA were generally the same as in the region‐based models. In brief, SVR was implemented using the sklearn “SVM” library with a precomputed linear kernel. A systematic hyperparameter search for C was implemented using the sklearn grid search method in a fivefold nested CV (stratified by age) over the search space 2^−7^, 2^−5^, 2^−3^, 2^−1^, 1, 2, 2^3^, 2^5^ and 2^7^ and the scoring parameter specified as negative MAE. RVR was implemented using the sklearn‐rvm library for expectation‐maximisation RVR with a precomputed linear kernel. GPR on voxel‐level data without PCA could not be performed due to computational restraints.

The 150 principal components from PCA (Section 2.3) were precomputed and then served as input to the voxel‐based SVR, RVR and GPR models with dimensionality reduction. The principal components were scaled using the robust scaler from the sklearn library. Linear SVR, RVR and GPR with PCA were implemented using the same libraries, functions and parameters as for the region‐based analysis.

#### 2.5.2 Cross‐validation

In this study, 10‐fold CV was performed to train each regression model on nine subsets of randomly selected subjects and then test the model on the one subset that was left out, also called the validation set. For all models, the CV training and validation sets were stratified by age to preserve the same age distribution in the training and validation set. To improve the replicability of the results, we repeated the 10‐fold CV 10 times (method called 10‐times 10‐fold CV) following the recommendations by Bouckaert and Frank (2004). This method resulted in 100 performance measures per model that were later used in our hypothesis tests.

#### 2.5.3 Model accuracy

The difference between the participant's predicted brain age and their chronological age was used to measure the models' predictions at an individual level. This measure is also known as brain age gap estimation (BrainAGE; Franke et al., 2010), brain‐predicted age difference (brain‐PAD; Cole et al., 2018) or brain age delta (Smith, Vidaurre, Alfaro‐Almagro, Nichols, & Miller, 2019). It is calculated as BrainAGE = predicted age—chronological age, where a positive BrainAGE indicates that the participant's brain age was predicted to be older than their chronological age and vice versa.

We reported the performance of each model as the MAE, which is the mean of the absolute values of BrainAGE across all samples in the validation or test set. Furthermore, as suggested by Cole, Franke, and Cherbuin (2019), we also reported the “weighted MAE”, where the MAE is presented as a ratio of the age interval of the data set. In the cross‐validation results, we divided the MAE by the age range of this validation set, and in the independent test set, we divided the MAE by the age range of the independent test set. The age interval in our train and test data sets was [47, 73], so the MAE was divided by 73–47 = 26 to obtain the weighted MAE. Weighting by the age range makes the comparison of the results to other studies using different age ranges more meaningful.

As additional measures, we examined the root mean squared error (RMSE), which is more sensitive to outliers than MAE, the correlation coefficient Pearson's r for chronological age and predicted age, and the prediction *R* ^2^. These measures are explained in more detail below. All performance metrics were reported as the mean across all 100 models from the CV iterations and repetitions.

It is important to note that the prediction *R* ^2^ (also called cross‐validation *R* ^2^ or *q* ^2^) presented here differs from the coefficient of determination *R* ^2^ that is typically reported in regression studies as the square of the correlation coefficient. We followed recommendations by Scheinost et al. (2019) of reporting Pearson's *r* in combination with prediction *R* ^2^ to reflect the error between predicted and observed values more accurately, while the coefficient of determination *R* ^2^ reflects the error between the predictions and their fit to the regression line (Alexander, Tropsha, & Winkler, 2015; Scheinost et al., 2019). Whilst standard *R* ^2^ indicates how much variation in the sample is accounted for in the model, prediction *R* ^2^ denotes the amount of variation in potential new observations that were not part of the model development that is accounted for in the model. Scheinost et al. (2019) demonstrated that prediction *R* ^2^ is less likely to overestimate prediction performance evaluated through CV, making it is less biased.

#### 2.5.4 Statistical comparison of regressor performance

To test for differences in the performance of the evaluated regressors, we used the version of the paired Student's *t* test corrected for the violation of the independence assumption from repeated *k*‐fold CV during model training (Bouckaert & Frank, 2004; Nadeau & Bengio, 2003). The regular paired Student's *t* test would have led to an increased probability of type I error, as first demonstrated by Dietterich (1998). The corrected version of the *t* test outputs the t statistic and p‐value for the comparison of two regressors with the null hypothesis that their performance is not statistically different (i.e., their performance difference is equal to 0).

This corrected version of the *t* test was used to assess whether the means of MAE values resulting from the 10‐times 10‐fold CV of two models were statistically different. The pairwise comparison of the eight models in this study (i.e., three applied to region‐level data, the two models applied to voxel‐level data without PCA, and the three applied to voxel‐level data with PCA) resulted in 28 combinations. Bonferroni correction for multiple comparisons was used to determine the significance level (α = .05/28 comparisons ≈ .0018). If a statistically significant difference was found between two models, the relatively lower/higher MAE of each model was used to infer whether a model performed better or worse than the other.

### 2.6 Model generalisation

We also tested model generalisation by applying the models to an independent test set, the Site 2 data from UK Biobank. Testing in an independent data set eliminates sample bias in the assessment of performance, and it provides a more realistic representation for the potential application of brain age prediction as a biomarker in clinical practice or similar scenarios. In such real‐world problems, the data for age prediction would likely come from several sources with confounding imaging effects, such as scanner hardware or operator inconsistencies. For the region‐level data, the 100 regressor models and scalers, obtained from the 10‐times 10‐fold CV, were loaded one by one. The test data were first scaled in the case of region‐level data or masked in the case of voxel‐level data (using the template's brain mask) and then the loaded regressor models were applied to predict the brain age of each subject in the Site 2 data set. The corrected version of the Student's *t* test was used to assess the statistical significance of performance differences between the regressor models. A Student's *t* test was also used to test for differences in the age distributions between Sites 1 and 2.

### 2.7 Covariate analysis of age

Chronological age may have a confounding effect on brain age prediction models (Le et al., 2018). Whilst we did not account for this potential effect in the models themselves, we assessed it in a post‐hoc analysis using Spearman's rank‐order correlation. The age‐BrainAGE correlation measure is also known as “age bias” (de Lange & Cole, 2020) and it can be used to assess whether BrainAGE needs to be corrected for chronological age (for a discussion, see Le et al., 2018). Spearman's rank‐order correlation is a nonparametric assessment of the monotonicity of the relationship between two variables, in this case chronological age and BrainAGE. Spearman's correlation coefficient (*r*_*S*_) describes the degree and direction of the relationship on a scale of −1 to 1 to indicate if the variables are negatively correlated, positively correlated or not at all correlated.

### 2.8 Analysis of training set size

We used bootstrapping to estimate the stability of the machine learning models for different training set sizes. Bootstrapping is a resampling method, where the original training set is resampled with replacement to obtain a new training set of the desired sample size. Bootstrap is commonly used in machine learning classification studies to assess the robustness of performance across training set sizes and determine the minimum training set size required for the model to performance above chance level (e.g., Nieuwenhuis et al., 2012). Therefore, we chose to systematically assess a wide range of training set sizes for each machine learning model to investigate how their MAE differed with smaller and larger training samples.

We created 1,000 bootstrap samples with replacement containing 54–1,080 subjects in the training set, with equal numbers of men and women per age group (starting at one man and one woman per age up to 20 each per age). This means that 54 subjects (27 men, 27 women) were added to the training set size in each iteration that may or may not overlap with the bootstrap training sample at the previous iteration. Additionally, we created a validation set containing 1,080 subjects (40 subjects per age, 20 women/20 men) that did not overlap with the subjects in the corresponding training set per iteration. Besides that, we used the whole Site 2 data set (with a non‐uniform age distribution) in order to assess the generalisation. For the voxel‐based models with PCA, we only assessed data sets with more than 150 subjects, because the PCA algorithm requires more samples than principal components. Furthermore, training set sizes above 500 were not calculated due the restriction in time and computer resources.

SVR, RVR and GPR models were retrained on each of the training bootstrap samples and the MAE was obtained in the corresponding validation bootstrap sample as well as the independent test set. To obtain the confidence interval (CI; 95% of confidence) for the estimates, we used the percentile method (Efron, 1981). We compared these models against a naïve approach where we used the data set mean age of the uniform distribution as the chance‐level prediction performance. In this case, the mean absolute distance between all chronological ages and the mean value is the standard deviation of the uniform distribution (i.e., $\sqrt{{\left(\mathrm{age}\text{\_}\max-\mathrm{age}\text{\_}\min\right)}^{2}/12}=\sqrt{{\left(73-47\right)}^{2}/12}=7.5$years). This approach was used to assess the sample size required for the bootstrap models to perform better than chance level, that is, if the confidence interval did not overlap with the chance value.

### 2.9 Experiments

All experiments were conducted in Python 3 using the scikit‐learn library for SVR and GPR (https://scikit-learn.org/stable/; Pedregosa et al., 2011), sklearn‐rvm library for RVR (https://github.com/Mind-the-Pineapple/sklearn-rvm), and statsmodels library (https://www.statsmodels.org/stable/index.html; Seabold & Perktold, 2010). The code is available at https://github.com/MLMH-Lab/Brain-age-prediction.

## 3 RESULTS

### 3.1 Model comparison

The results from the 10‐times 10‐fold CV of the SVR, RVR and GPR models on region‐level data and voxel‐level data (with or without PCA) are summarised in Table 2, whereas the statistical significance of the model comparisons is reported in Table 3. The models achieved MAE values between 3.69 years (voxel‐based RVR without PCA) to 4.43 years (region‐based SVR and RVR; Table 2). Overall, the voxel‐based models with PCA performed significantly better than all other models (Table 3). The performance of the three voxel‐based models with PCA were very similar with MAE of ~3.9 years, and the performance of the region‐based models was also highly similar across all measures with MAE of ~4.4 years. This was less consistent for the voxel‐based models without PCA. Whilst the lowest MAE was actually achieved by voxel‐based RVR without PCA, this was not statistically different from any other models. The latter is probably due to its high standard deviations. Additional analysis revealed that 41 out of the 100 model iterations underfitted to the training data with <600 relevance vectors (data not shown), which is likely because the relevance vector selection threshold during training was too low. The voxel‐based SVR without PCA performed worse than its RVR counterpart with an MAE value of 4.33 years, though this difference is not statistically significant. The higher RMSE values for all models indicated the presence of a few outliers in the sample.

**TABLE 2:** Performance metrics for region‐ or voxel‐based SVR, RVR and GPR models in 10‐times 10‐fold CV (UK Biobank Site 1) with or without dimensionality reduction through PCA

Data type	Method	MAE	Weighted MAE	RMSE	Pearson's *r*	Prediction *R* ^2^	Age‐BrainAGE correlation
Region	SVR	4.43 (0.09)	0.17	5.48 (0.12)	0.62 (0.00)	0.37 (0.03)	−0.73 (0.00)
	RVR	4.43 (0.09)	0.17	5.44 (0.11)	0.62 (0.00)	0.38 (0.02)	−0.78 (0.00)
	GPR	4.42 (0.09)	0.17	5.44 (0.11)	0.62 (0.00)	0.38 (0.02)	−0.77 (0.00)
Voxel (no PCA)	SVR	4.33 (0.10)	0.17	5.43 (0.12)	0.73 (0.00)	0.39 (0.03)	−0.23 (0.00)
	RVR	3.69 (0.45)	0.14	4.60 (0.50)	0.75 (0.02)	0.55 (0.11)	−0.62 (0.02)
Voxel (PCA)	SVR	3.89 (0.08)	0.15	4.86 (0.11)	0.71 (0.00)	0.51 (0.02)	−0.68 (0.00)
	RVR	3.90 (0.08)	0.15	4.85 (0.10)	0.71 (0.00)	0.51 (0.02)	−0.72 (0.00)
	GPR	3.90 (0.08)	0.15	4.85 (0.10)	0.71 (0.00)	0.51 (0.02)	−0.71 (0.00)

*Note*: . In each column, the data are presented as mean value (*SD*) across all model iterations. GPR performance on voxel‐level data without PCA was not assessed.

**TABLE 3:** Statistical assessment of differences in model performance in terms of MAE of the region‐ or voxel based SVR, RVR and GPR in 10‐times 10‐fold CV

	SVR (region)	RVR (region)	GPR (region)	SVR (voxel, no PCA)	RVR (voxel, no PCA)	SVR (voxel, PCA)	RVR (voxel, PCA)	GPR (voxel, PCA)
SVR (region)	–	0.98 (−0.02)	0.80 (0.26)	0.50 (0.68)	0.14 (1.50)	**<0.001 (5.11)**	**<0.001 (5.29)**	**<0.001 (5.31)**
RVR (region)		–	0.48 (0.70)	0.48 (0.71)	0.13 (1.51)	**<0.001 (5.33)**	**<0.001 (5.54)**	**<0.001 (5.57)**
GPR (region)			–	0.51 (0.67)	0.14 (1.50)	**<0.001 (5.27)**	**<0.001 (5.48)**	**<0.001 (5.52)**
SVR (voxel, no PCA)				–	0.20 (1.30)	**<0.001 (3.58)**	**<0.001 (3.35)**	**<0.001 (3.41)**
RVR (voxel, no PCA)					–	0.69 (−0.40)	0.66 (−0.44)	0.67 (−0.43)
SVR (voxel, PCA)						–	0.44 (0.78)	0.31 (1.02)
RVR (voxel, PCA)							–	0.58 (−0.55)
GPR (voxel, PCA)								–

*Note*: The table presents the *p*‐values (*t*‐statistic). Statistical significance was assessed using a version of the paired Student's *t* test corrected for the violation of the independence assumption in CV. The significance level was corrected for multiple comparisons using Bonferroni's method (α = .05/28 ≈ .0018). Statistically significant differences between model performances are shown in bold.

Chronological age and predicted age showed moderate positive correlations for all models (*r* ~ .6 for region‐based models and *r* ~ .7 for voxel‐based models). The prediction *R* ^2^ values showed a similar pattern of moderate positive scores that are slightly higher for voxel‐based models than for region‐based models (*R* ^2^ ~ .4 for region‐based models and *R* ^2^ ~ .5 for voxel‐based models) with the exception of voxel‐based SVR without PCA (*R* ^2^ ~ .4). This means that the models would account for 40–50% of variance in new data observations.

### 3.2 Model generalisation

As presented in Table 4, applying the regressor models to an independent data set, UK Biobank Site 2, led to MAE scores ~4.1 years for all region‐based models and ~ 3.8 years for the voxel‐based models with PCA. The latter models with PCA performed better than the region‐based ones (Table 5). In terms of MAE, voxel‐level RVR without PCA had the best performance with 3.66 years; however, this was not statistically different from any other models due to its high *SD*. The voxel‐based SVR model without PCA performed worst with a MAE of 4.69 years, which was worse than the models with PCA and all region‐based models. The correlation between chronological and predicted age was moderate to high for all models (*r* ~ .7). Similarly, the prediction R^2^ scores were moderate for most models with slightly higher values for the voxel based models (*R* ^2^ ~ .5) with the exception of voxel‐based SVR (*R* ^2^ = .21), which is in line with the other worse performance measures for this model.

**TABLE 4:** Performance metrics of region‐ or voxel‐based SVR, RVR and GPR models with or without PCA in independent test set (UK Biobank Site 2)

Data type	Method	MAE	Weighted MAE	RMSE	Pearson's *r*	Prediction *R* ^2^	Age‐BrainAGE correlation
Region	SVR	4.06 (0.02)	0.16	5.07 (0.02)	0.65 (0.00)	0.42 (0.00)	−0.72 (0.00)
	RVR	4.10 (0.02)	0.16	5.06 (0.02)	0.66 (0.00)	0.42 (0.00)	−0.77 (0.00)
	GPR	4.08 (0.01)	0.16	5.05 (0.01)	0.66 (0.00)	0.42 (0.00)	−0.77 (0.00)
Voxel (no PCA)	SVR	4.69 (0.09)	0.18	5.92 (0.11)	0.71 (0.01)	0.21 (0.03)	−0.16 (0.01)
	RVR	3.66 (0.57)	0.14	4.51 (0.61)	n/a^a^	0.53 (0.15)	−0.82 (0.16)
Voxel (PCA)	SVR	3.77 (0.04)	0.15	4.65 (0.04)	0.74 (0.00)	0.51 (0.01)	−0.60 (0.01)
	RVR	3.82 (0.03)	0.15	4.65 (0.04)	0.74 (0.00)	0.51 (0.01)	−0.64(0.01)
	GPR	3.81 (0.03)	0.15	4.64 (0.04)	0.74 (0.00)	0.51 (0.01)	−0.63 (0.01)

*Note*: In each column, the data are presented as mean value (*SD*) of the predictions from the 100 model iterations. GPR performance on voxel‐level data without PCA was not assessed.

^a^ Pearson's *r* for voxel‐based RVR without PCA could not be calculated, since the model underfitted to the training set and predicted the sample mean age in 41 out of the 100 iterations; therefore, their predictions in the independent test set had no variance.

**TABLE 5:** Statistical assessment of differences in model performance in terms of MAE of the region‐ or voxel‐based SVR, RVR and GPR models in an independent test set (UK Biobank Site 2)

	SVR (region)	RVR (region)	GPR (region)	SVR (voxel, no PCA)	RVR (voxel, no PCA)	SVR (voxel, PCA)	RVR (voxel, PCA)	GPR (voxel, PCA)
SVR (region)	‐	0.04 (−2.07)	0.15 (−1.46)	**<0.001 (−6.23)**	0.51 (0.66)	**<0.001 (6.80)**	**<0.001 (6.62)**	**<0.001 (6.72)**
RVR (region)		‐	0.23 (1.21)	**<0.001 (−5.92)**	0.47 (0.73)	**<0.001 (7.80)**	**<0.001 (7.82)**	**<0.001 (7.76)**
GPR (region)			‐	**<0.001 (−6.10)**	0.49 (0.70)	**<0.001 (7.38)**	**<0.001 (7.58)**	**<0.001 (7.65)**
SVR (voxel, no PCA)				‐	0.10 (1.66)	**<0.001 (8.94)**	**<0.001 (8.58)**	**<0.001 (8.56)**
RVR (voxel, no PCA)					‐	0.86 (−0.18)	0.80 (−0.25)	0.81 (−0.25)
SVR (voxel, PCA)						‐	0.10 (1.67)	0.08 (−1.77)
RVR (voxel, PCA)							‐	0.66 (0.44)
GPR (voxel, PCA)								‐

*Note*: The table presents the *p*‐values (*t*‐statistic). Statistical significance was assessed using a version of the paired Student's *t* test corrected for the violation of the independence assumption in CV. The significance level was corrected for multiple comparisons using Bonferroni's method (α = .05/28 ≈ .0018). Statistically significant differences between model performances are shown in bold.

Importantly, compared to Table 2, all models except for voxel‐level SVR without PCA showed better performance in the independent test set than in the CV (significance not assessed), suggesting that they generalised well. The Student's *t* test indicated that the age distribution was statistically significantly different between Sites 1 and 2 (*p* < .001) with the Site 2 data set being slightly older.

### 3.3 Covariate analysis of age

In the CV evaluation of the models, a high negative Spearman's correlation coefficient (r_S_) for chronological age and BrainAGE was found for all models (*r*_S_ *≈* −.7) except for voxel‐based SVR, where a low negative association was found (*r*_S_ = −.23; Table 2). Similarly, in the generalisation analysis, the models also displayed high negative age‐BrainAGE correlations for all models (*r*_S_ *≈* −.7) except for voxel‐based SVR without PCA (*r*_S_ = −.16; Table 4).

### 3.4 Analysis of training set size

**FIGURE 1:**
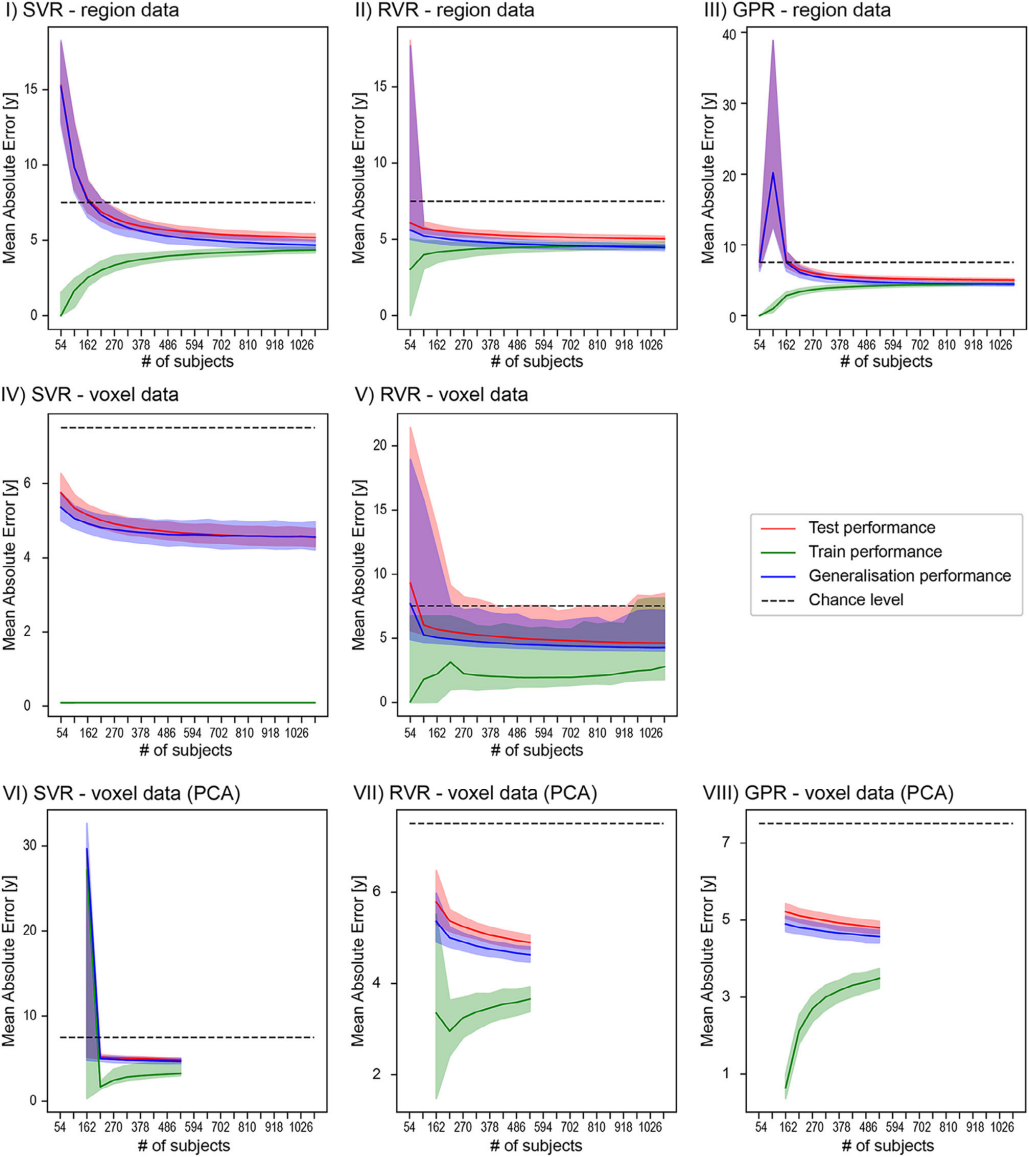
MAE of region‐ and voxel‐based SVR, RVR, and GPR models with or without PCA for the training set size compared to chance level (7.5 years; black dotted line). MAE is shown for the performance within the training (red line) and test set (green line) of the CV (Site 1) and in the independent test set (Site 2; blue line). The confidence intervals (shaded areas) for the different size of the data sets were calculated using bootstrap analysis. Note that bootstrap training samples were selected to be age‐ and sex‐homogeneous of increasing size with the minimum of one man and one woman per age and maximum of 20 men and 20 women per age. For the voxel‐based models with PCA, data sets with <150 subjects could not be assessed, because the PCA algorithm requires more samples than principal components. Furthermore, training set sizes above 500 were not calculated due limited time and computational resources

The analysis of training set size showed that the performance of the regression models in an independent test set improved with larger training set size, but the minimum number of subjects required for performance above chance level varied with model type (Figure 1). Among the region‐based models, RVR required about 120 subjects to perform above chance level in an independent data set, while the SVR and GPR models trained on the same data needed more than 270 subjects to achieve the same performance. However, the MAE for GPR increased sharply between 54 and 108 subjects before decreasing again at 152, probably due to overfitting at small sample sizes. To summarise, RVR appeared to require less than half the sample size than SVR or GPR to predict brain age on region‐level data better than chance.

The MAE performance for voxel‐level data showed different patterns for increasing training set sizes. In the absence of PCA, the training performance of the SVR model was close to zero for any training set size, because almost all training samples were assigned to support vectors. In contrast, many of the RVR algorithm repetitions underfitted, leading to very broad confidence intervals for all types of performance assessments and training set sizes. The voxel‐based SVR model with PCA needed a minimum of approximately 200 subjects to perform above chance, whilst RVR and GPR with PCA performed above chance level for all training set sizes tested.

## 4 DISCUSSION

**FIGURE 2:**
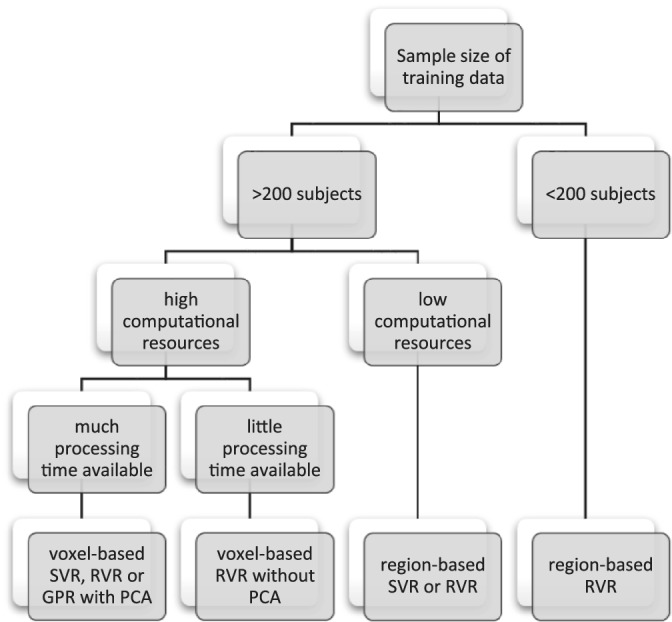
A decision tree for researchers choosing the most suitable brain age prediction model for their project. The ranking is inferred from our experience developing the models as well as the results of our investigation. These recommendations are thus built on the UK Biobank data set and our specific computational resources, so any application to other projects should be done with caution. The models in this study were developed using a high‐end consumer‐grade desktop computer with a 16‐core (32‐processes) CPU @ 3.40 GHz utilising 128 GB RAM. The voxel‐based models with PCA took 1–2 weeks to train, while the voxel‐based models without PCA took <1 day. The region‐based models took <1 hr to train

The present study compared SVR, RVR and GPR with different morphometric input to perform brain age prediction. A total of eight models was assessed. The wide range of methods used in previous studies makes it challenging to disentangle the direct effect of model choice and other factors, such as the characteristics of the data set. In our study, we showed that the type of data input is generally more important than the choice of model, but various other aspects like data set size and processing time available should be considered when choosing a model. In Figure 2, we provide a decision tree that may help inform the model choice. This decision tree is based on the sequence of steps a researcher would typically take when designing a brain age study and is informed by the results of the present investigation. It is important to note that these results, and therefore our recommendations, are based on the UK Biobank. For example, our recommendations regarding the sample size and computational resources may be dependent on the characteristics of this specific data set. However, we believe that the general idea that some models require considerably more training data and computational resources than others can be generalised to other data sets.

Based on the literature, our first hypothesis was that all models would perform with MAE values below 5 years. With scores ranging from 3.7 to 4.7 years in the CV as well as the independent test set, this hypothesis was confirmed. These findings are generally in line with existing studies using a comparable setup, where MAE values in CV and independent data sets tend to fall between 3.9 and 6.2 years and 4.8 and 7.1 years, respectively (see Table S1 for an overview of related studies).

Our models showed moderate‐high positive associations between age and predicted age (*r* ≈ .7 for all models) and they accounted for 40–50% of variation in new data (prediction *R* ^2^ ≈ .4–.5). Whilst these values are relatively high, the associations were lower than previous brain age studies that reported *r* values above .9 (Cole, Leech, & Sharp, 2015; Franke et al., 2010; Gutierrez Becker, Klein, & Wachinger, 2018; Kondo et al., 2015). The latter studies have in common that they covered a wider age range, including young people. In these age groups, the ongoing brain maturational changes make the task of brain age prediction easier. It therefore is possible that the limited and older age range in our sample along with the greater heterogeneity because of our unique data set size contributed to the lower—though still relatively high—*r* values of our models.

Our second hypothesis was that region‐based models would outperform voxel‐level ones due to the “curse of dimensionality” and high level of redundancy in the latter data, for example, high spatial correlations between voxels. This hypothesis was not confirmed, as there was no significant difference between the region‐ and voxel‐based models (without PCA) in CV. Nonetheless, it appeared that dimensionality reduction through PCA could successfully remove redundancy to the extent that voxel‐based models with PCA performed significantly better than the region‐based models. This finding suggests that some of the age‐related heterogeneity might be lost if the MRI data are summarised as regional volumes using FreeSurfer software. One previous study compared region‐ and voxel‐level data input for GPR, but there was no clear difference in performance based on data type only (Gutierrez Becker et al., 2018). Comparing previous studies using either region‐ or voxel‐level data as input also does not point at either type of data preprocessing being more suited for brain age prediction using SVR, RVR or GPR (Table S1).

Our third hypothesis was that RVR would perform best regardless of data input type, because it is seen as the “most popular” algorithm for brain age prediction (Cole et al., 2019). This hypothesis was not confirmed. Although voxel‐based RVR without PCA showed the lowest MAE overall with ~3.7 years, the difference to the other models was not statistically significant due to its high variance. The analysis of training set size also suggested that many iterations underfitted to the training set, which likely caused this variance. Therefore, we cannot conclude that RVR is the best model choice for brain age prediction regardless of data input. Previous studies on RVR or SVR that did not show a clear superior model (Table S1). Only two studies seem to have directly compared these two methods. For example, in Kondo et al. (2015), RVR performed slightly better than SVR in terms of MAE (4.50 and 4.73 years after dimensionality reduction, respectively). In Franke et al. (2010), RVR also performed slightly better than SVR after dimensionality reduction (4.98 vs. 5.10 after dimensionality reduction) but not without dimensionality reduction (5.23 vs. 5.14 without dimensionality reduction). This coincided with our findings, where PCA improved SVR but not RVR performance. However, neither of the previous studies assessed the significance of the difference, and we did not find a statistically significant difference between SVR and RVR if trained on the same data.

In terms of the GPR model, performance did not differ to SVR and RVR if trained on the same data. This confirms findings from a previous study where RVR and GPR were compared (Aycheh et al., 2018). However, there is little data available on this comparison and especially GPR on region‐based data seems to be rare in the brain age literature. Our region‐based GPR model had a smaller MAE than Gutierrez Becker et al. (2018), but higher than Aycheh et al. (2018). The MAE of the voxel‐based GPR model with PCA is lower than previous comparable models by >1 year (Cole et al., 2015, 2018; Monté‐Rubio, Falcón, Pomarol‐Clotet, & Ashburner, 2018; Table S1).

While MAE values of our models were generally low, their weighted MAE scores of 0.14 and above were notably higher than in other studies on SVR, RVR and GPR, where the scores tend to fall between 0.07 and 0.09 (Table S1). This is likely due to the smaller age range used here, as detailed in the limitations below. Although weighted MAE has not been formally validated as a measure of model performance, taking into account the age range of the training and test set is a useful exercise. A potential reason for the relatively high weighted MAE scores in our study might be greater heterogeneity in our sample due to the very large data set of >10,000 subjects, while the largest comparable study had around 3,000 subjects (Valizadeh et al., 2017). The acquisition of 10,000 subjects in one scanner will likely take place over a much longer time period than smaller data sets, so the acquired images will also be affected by changes in the scanner environment. These scanner effects might further contribute to the heterogeneity of our sample. In short, while the large data set is a clear strength of our study, it might compromise the comparability of our results to other studies in terms of weighted MAE.

Our models showed relatively high negative correlations between chronological age and BrainAGE in the CV iterations as well as the independent test set (approx. −0.7 for all with the exception of voxel‐based SVR without PCA). This finding suggests that the models were equally and highly affected by regression to the mean (Le et al., 2018), although it is unclear why voxel‐based SVR may be less affected by this. Whilst the high confounding effect of chronological may be seen as a limitation of our study, we believe it does not affect the direct comparison of models, which was our primary objective. Nonetheless, future studies should revisit these models and include the correction for age in the training. Various types of correction have been proposed in recent years (Beheshti, Nugent, Potvin, & Duchesne, 2019; Cole et al., 2020; de Lange & Cole, 2020; Le et al., 2018).

In a clinical context, it is crucial for a model to generalise to data from different scanners, because the parameters and environment of a scanner can introduce considerable bias. It is important to note that the independent data set in the present study was acquired on a different scanner with the same acquisition parameters, so future studies should address how our models would perform in other independent data sets acquired using different scanners and acquisition parameters. Our models generalised well to the independent test set. Indeed, the region‐based models or the voxel‐level models with PCA performed slightly better in the independent data set than in the CV set by approx. 0.3 and 0.1 years, respectively. Statistical significance between CV and generalisation performance was not assessed. These findings suggest the promise of these models for real‐world application. In previous brain age prediction studies that compared model performance in an independent data set against the CV test, the models usually performed worse in the former (Cole et al., 2018; Franke et al., 2010; Lancaster, Lorenz, Leech, & Cole, 2018; Liem et al., 2017; see Table S1). However, similar to our findings, two studies also showed comparable performance in both (Cole et al., 2015; Le et al., 2018). Performance differences in an independent data set can likely be explained by sample characteristics, such as the similarity of this sample and the training data. In our case, the independent test set was acquired using the same acquisition protocol on a different scanner and the subjects came from the same population as the CV set. Noise and homogeneity should thus be similar between the samples. However, the independent test set appeared to be significantly older and it contained a slightly higher proportion of women (57%, see Table 1). So far, it is unclear whether sex has a considerable effect on brain age prediction, but this factor may have contributed to the performance differences between the sites in our study.

As expected, the analysis of training set size showed that larger sample sizes generally led to better prediction performances, though MAE scores did plateau with increasing training set size. For the region‐based models, RVR required only half the training sample size than the other two to make predictions better than chance level, suggesting its suitability for studies where the sample size is limited. The analysis of training set size for GPR showed a sharp decrease in performance (i.e., higher MAE) at the smaller training set sizes, which might indicate overfitting to the training sample in the smaller samples. To our knowledge, no other studies have systematically evaluated the impact of training set size analysis to brain age prediction. In one case, Franke et al. (2010) assessed the effect of training set size by running separate RVR models on the full training data set (*N* = 410), half the data set (*N* = 205) and a quarter of the data set (*N* = 103). The MAE decreased from the smallest training set (5.6 years) to the largest set (4.9 years), which coincided with our results.

While our investigation was based on healthy brain ageing, it is important to ponder the potential implications of our findings for studies in clinical populations. One of the most promising uses of brain age prediction is its relevance and use as a biomarker. It could, for example, be implemented as an individualised marker of brain health in diagnostic tools. The main idea is to quantify the deviation between predicted and chronological age. Brains that are predicted to be older than their true age might suggest aberrant age‐related changes and be associated with disease (Cole & Franke, 2017). Previous studies have assessed BrainAGE in various neurological and psychiatric disorders and they demonstrated that different stages of Alzheimer's disease as well as schizophrenia can present as accelerated ageing in the brain (Franke et al., 2010; Franke & Gaser, 2012; Gaser et al., 2013; Kaufmann et al., 2019; Koutsouleris et al., 2014, 2015; Nenadić et al., 2017; Schnack et al., 2016). One of the necessary characteristics of a biomarker is its reliability. Therefore, future studies could adopt a longitudinal design to (a) further examine the reliability of the brain age prediction methods through test–retest setups in single or multi‐scanner experiments, (b) learn more about the brain changes in health and disease, and (c) explore if brain age is a useful marker of treatment success in clinical trials.

The present study had three main limitations. First, whilst our data set size was quite large, the age range of 47–73 was smaller than most studies in the literature (e.g., Ashburner, 2007; Cole et al., 2018, Cole et al., 2015; Franke et al., 2010; Gutierrez Becker et al., 2018; Le et al., 2018; Madan & Kensinger, 2018; Wang et al., 2014; Table S1). Furthermore, we excluded non‐white ethnicities from the analysis because of data availability. These two factors imply that our models cannot be applied to data sets with ages or ethnicities that were not included in the training sample. Second, whilst the present study explored a wide range of methodological choices in terms of machine learning models and data input, there are several other methods that could be assessed in the future. For example, we did not explore nonlinear regression models, because we were interested in the interpretability of the models. Nevertheless, Ashburner (2007) directly compared the performance of RVR using either a linear and radial‐basis kernel and found performance improvements with some configurations of the nonlinear one, so this seems to be an interesting area for future research. In addition, deep convolutional neural networks have shown to have a high accuracy when predicting brain age (Cole et al., 2017; Ito et al., 2018; Peng, Gong, Beckmann, Vedaldi, & Smith, 2021). Third, the present study was based on the use of a single neuroimaging modality. Our models could likely be improved by using multimodal input data. Previous research has shown that even combining different morphometric features, such as cortical thickness, surface area and/or curvature information, can improve model performance (Valizadeh et al., 2017; Wang et al., 2014; Zhao et al., 2018), because they may carry potentially complementary information about brain age. Similarly, Gutierrez Becker et al. (2018) achieved better performance of their GPR model when combining voxel‐level and region‐level features than looking at them separately, and Liem et al. (2017) were the first to combine structural and functional MRI in brain age prediction to achieve better performance. Multimodal data sets could also integrate conventional health assessments of ageing, which might improve the performance and generalisation of the models, making them a promising avenue for future brain age research (Cole et al., 2018).

## 5 CONCLUSION

By systematically and rigorously comparing the performance of different algorithms on the same data set, the present study demonstrated that SVR, RVR and GPR models are suitable for brain age prediction based on both region‐ and voxel‐based morphometric data. When designing a brain age study, researchers should consider various factors to choose the most appropriate model. Most importantly, while the overall best performance was achieved by voxel‐based models with dimensionality reduction through PCA, this was also the most computationally expensive approach and might not be feasible if computational or time resources are limited. Furthermore, neuroimaging studies are often limited in their sample size. Our analysis of training set size revealed that region‐based RVR required the smallest training set to yield good performance with about 120 training subjects. This RVR model was also the simplest and fastest to implement. In conclusion, by providing clarification on important methodological aspects, the present investigation represents a step towards achieving the full clinical potential of brain age prediction, which lies in its application to the diagnosis, prognosis and monitoring of brain disorders. We are making all of our scripts open source (available at https://github.com/MLMH-Lab/Brain-age-prediction) in the hope that this will aid future research.

## CONFLICT OF INTERESTS

The authors declare no conflict of interest.

## AUTHOR CONTRIBUTIONS

Lea Baecker: data curation, methodology, region‐based analysis, writing. Jessica Dafflon: voxel‐based analysis, review & editing. Pedro F. da Costa: voxel‐based analysis, review & editing. Rafael Garcia‐Dias: data curation, review & editing. Sandra Vieira: data curation, review & editing. Cristina Scarpazza: data curation, review & editing. Vince D. Calhoun: funding acquisition, review & editing. João R. Sato: funding acquisition, review & editing. Andrea Mechelli: funding acquisition, review & editing. Walter H. L. Pinaya: conceptualisation, data curation, methodology, region‐ and voxel‐based analysis, review & editing.

## Supporting information


**Appendix S1:** Supporting information

## Data Availability

The data used for this work was obtained from the UK Biobank Resource (Project Number 40323). Due to the nature of the data sharing agreement, we are not allowed to publish the data. The code in this article is publicly available at https://github.com/MLMH-Lab/Brain-age-prediction.
