# Supplementary material for: Brain age prediction: A comparison between machine learning models using region‐ and voxel‐based morphometric data
Source: Hum Brain Mapp. 2021 Mar 19;42(8):2332–46. doi: 10.1002/hbm.25368 (PMC8090783; doi:10.1002/hbm.25368)
Supplement: Supplementary file 1 — Appendix S1: Supporting information [file HBM-42-2332-s001.docx]

**Supplementary materials**

*Table S1: Comparison of our prediction results to comparable region- and voxel-based brain age prediction models using SVR, RVR or GPR in healthy subjects in the literature. Where multiple similar models were studied, the best performance was reported in the table. The weighted MAE for all studies was calculated as described in section 2.5.3, because most studies did not include this measure originally. The label ‘region’ for data preprocessing includes studies on regional volumes as well as cortical thickness.*

*Abbreviations: CV, cross-validation; GM, grey matter; GPR, Gaussian process regression; HC, healthy controls; MAE, mean absolute error; PCA, principal component analysis; RMSE, root mean squared error; RVR, relevance vector regression; SVR, support vector regression; WM, white matter*

|  |  |  |  | **CV** | | | | | **Independent test set** | | | | |
| --- | --- | --- | --- | --- | --- | --- | --- | --- | --- | --- | --- | --- | --- |
| **Author** | **Model** | **Data pre- processing** | **Dimensionality reduction** | **N** | **Age range** | **MAE** | **Weighted MAE** | **RMSE** | **N** | **Age range** | **MAE** | **Weighted MAE** | **RMSE** |
| Ashburner (2007) | RVR | voxel | - | 471 | 17-79 | - | - | 6.50 | - | - | - | - | - |
| Franke et al. (2010) | RVR | voxel | PCA | 547 | 19-86 | 4.61 | 0.07 | 5.90 | 108 | 20-59 | 5.44 | 0.14 | 6.73 |
|  | RVR | voxel | - | 547 | 19-86 | 4.96 | 0.07 | - | 108 | 20-59 | 5.57 | 0.14 | - |
|  | SVR | voxel | PCA | 547 | 19-86 | 4.85 | 0.07 | - | 108 | 20-59 | 5.42 | 0.14 | - |
|  | SVR | voxel | - | 547 | 19-86 | 4.85 | 0.07 | - | 108 | 20-59 | 5.51 | 0.14 | - |
| Koutsouleris et al. (2014) | SVR | voxel | PCA | 800 | 18-65 | 4.60 | 0.10 | - | - | - | - | - | - |
| Su et al. (2013) | RVR | voxel | sparse representation | 290 / 84 | 18-91 / 19-79 | 5.7 / 4.7 | 0.08 /  0.08 | - | - | - | - | - | - |
| Wang et al. (2014) | RVR | region | - | 360 | 20-82 | 4.57 | 0.07 | 5.57 | - | - | - | - | - |
| Kondo et al. (2015) | RVR | region | - | 1146 | 20-75 | 4.63 | 0.08 | 5.81 | - | - | - | - | - |
|  | RVR | region | repeated removal of one | 1146 | 20-75 | 4.50 | 0.08 | 5.65 | - | - | - | - | - |
|  | SVR | region | - | 1146 | 20-75 | 5.11 | 0.09 | 6.42 | - | - | - | - | - |
|  | SVR | region | repeated removal of one | 1146 | 20-75 | 4.73 | 0.09 | 5.99 | - | - | - | - | - |
| Cole et al. (2015) | GPR | voxel (GM) | - | 1537 | 18-90 | 6.20 | 0.09 | - | 113 | - | 5.80 | - | - |
|  | GPR | voxel (WM) | - | 1537 | 18-90 | 6.16 | 0.09 | - |  | - | 6.35 | - | - |
| Fujimoto et al. (2016) | RVR | region | repeated removal of one | 1099 | 20-80 | 4.48 | 0.07 | 5.74 | - | - | - | - | - |
| Liem et al. (2017) | SVR | region | - | 2354 | 19-82 | 4.83 | 0.08 | - | 475 | 18-85 | 7.39 | 0.11 | - |
| Valizadeh et al. (2017) | SVR | region | - | 3144 | 8-96 | - | - | - | 1572 | 8-18 | 1.14 | - | - |
|  |  |  |  |  |  |  |  |  |  | 18-65 | 5.65 | - | - |
|  |  |  |  |  |  |  |  |  |  | 65-96 | 7.04 | - | - |
| Aycheh et al. (2018) | RVR | region | - | 2705 | 45-91 | 4.13 | 0.09 | 5.24 | - | - | - | - | - |
|  | RVR | region | sparse group Lasso | 2705 | 45-91 | 4.09 | 0.09 | 5.19 | - | - | - | - | - |
|  | GPR | region | - | 2705 | 45-91 | 4.08 | 0.09 | 5.18 | - | - | - | - | - |
|  | GPR | region | sparse group Lasso | 2705 | 45-91 | 4.05 | 0.09 | 5.16 | - | - | - | - | - |
| Cole et al. (2018) | GPR | voxel | - | 2001 | 19-90 | 5.02 | 0.07 | 6.31 | 669 | 72-73 | 7.08 | 7.08 | 8.85 |
| Gutierrez Becker et al. (2018) | GPR | region | - | 1543 | 6-92 | 5.52 | 0.06 | - | - | - | - | - | - |
|  | GPR | region | - | 1543 | 6-92 | 6.50 | 0.08 | - | - | - | - | - | - |
|  | GPR | voxel | PCA | 1543 | 6-92 | 5.65 | 0.07 | - | - | - | - | - | - |
|  | GPR | region + voxel | PCA - voxel | 1543 | 6-92 | 3.86 | 0.04 | - | - | - | - | - | - |
| Lancaster et al. (2018) | SVR | voxel | - | 2003 | 16-90 | 5.08 | 0.07 | - | 648 | 18-88 | 6.08 | 0.09 | - |
| Le et al. (2018) | SVR | region | - | 475 | 18-60 | 5.10 | 0.12 | - | 489 (incl. non-HC) | 18-56 | 4.84 | 0.13 | - |
| Madan & Kensinger (2018) | RVR | region | PCA | 1056 | 18-94 | - | - | - | 176 / 116 | 18-83 / 20-87 | 6-7 median | - | - |
| **Our models** | **SVR** | **region** | **-** | **10480** | **47-73** | **4.43** | **0.17** | **5.48** | **334** | **47-73** | **4.06** | **0.16** | **5.07** |
|  | **RVR** | **region** | **-** | **10480** | **47-73** | **4.43** | **0.17** | **5.44** | **334** | **47-73** | **4.10** | **0.16** | **5.06** |
|  | **GPR** | **region** | **-** | **10480** | **47-73** | **4.42** | **0.17** | **5.44** | **334** | **47-73** | **4.08** | **0.16** | **5.05** |
|  | **SVR** | **voxel** | **-** | **10480** | **47-73** | **4.33** | **0.17** | **5.43** | **334** | **47-73** | **4.69** | **0.18** | **5.92** |
|  | **RVR** | **voxel** | **-** | **10480** | **47-73** | **3.69** | **0.14** | **4.60** | **334** | **47-73** | **3.66** | **0.14** | **4.51** |
|  | **SVR** | **voxel** | **PCA** | **10480** | **47-73** | **3.89** | **0.15** | **4.86** | **334** | **47-73** | **3.77** | **0.15** | **4.65** |
|  | **RVR** | **voxel** | **PCA** | **10480** | **47-73** | **3.90** | **0.15** | **4.85** | **334** | **47-73** | **3.82** | **0.15** | **4.65** |
|  | **GPR** | **voxel** | **PCA** | **10480** | **47-73** | **3.90** | **0.15** | **4.85** | **334** | **47-73** | **3.81** | **0.15** | **4.64** |

*Table S2: List of excluded diagnoses of brain-based disorders based on UK Biobank data code 19 'ICD10'. Further information is available on the UK Biobank website (https://biobank.ctsu.ox.ac.uk/crystal/coding.cgi?id=19&nl=1).*

| Code | Name |
| --- | --- |
| F00 | F00 Dementia in Alzheimer's disease |
| F000 | F00.0 Dementia in Alzheimer's disease with early onset |
| F001 | F00.1 Dementia in Alzheimer's disease with late onset |
| F002 | F00.2 Dementia in Alzheimer's disease |
| F009 | F00.9 Dementia in Alzheimer's disease |
| F01 | F01 Vascular dementia |
| F010 | F01.0 Vascular dementia of acute onset |
| F011 | F01.1 Multi-infarct dementia |
| F012 | F01.2 Subcortical vascular dementia |
| F013 | F01.3 Mixed cortical and subcortical vascular dementia |
| F018 | F01.8 Other vascular dementia |
| F019 | F01.9 Vascular dementia |
| F02 | F02 Dementia in other diseases classified elsewhere |
| F020 | F02.0 Dementia in Pick's disease |
| F021 | F02.1 Dementia in Creutzfeldt-Jakob disease |
| F022 | F02.2 Dementia in Huntington's disease |
| F023 | F02.3 Dementia in Parkinson's disease |
| F024 | F02.4 Dementia in human immunodeficiency virus [HIV] disease |
| F028 | F02.8 Dementia in other specified diseases classified elsewhere |
| F03 | F03 Unspecified dementia |
| F04 | F04 Organic amnesic syndrome |
| F05 | F05 Delirium |
| F050 | F05.0 Delirium not superimposed on dementia |
| F051 | F05.1 Delirium superimposed on dementia |
| F058 | F05.8 Other delirium |
| F059 | F05.9 Delirium |
| F06 | F06 Other mental disorders due to brain damage and dysfunction and to physical disease |
| F060 | F06.0 Organic hallucinosis |
| F061 | F06.1 Organic catatonic disorder |
| F062 | F06.2 Organic delusional [schizophrenia-like] disorder |
| F063 | F06.3 Organic mood [affective] disorders |
| F064 | F06.4 Organic anxiety disorder |
| F065 | F06.5 Organic dissociative disorder |
| F066 | F06.6 Organic emotionally labile [asthenic] disorder |
| F067 | F06.7 Mild cognitive disorder |
| F068 | F06.8 Other specified mental disorders due to brain damage and dysfunction and to physical disease |
| F069 | F06.9 Unspecified mental disorder due to brain damage and dysfunction and to physical disease |
| F07 | F07 Personality and behavioural disorders due to brain disease |
| F070 | F07.0 Organic personality disorder |
| F071 | F07.1 Postencephalitic syndrome |
| F072 | F07.2 Postconcussional syndrome |
| F078 | F07.8 Other organic personality and behavioural disorders due to brain disease |
| F079 | F07.9 Unspecified organic personality and behavioural disorder due to brain disease |
| F09 | F09 Unspecified organic or symptomatic mental disorder |
| F10 | F10 Mental and behavioural disorders due to use of alcohol |
| F100 | F10.0 Acute intoxication |
| F101 | F10.1 Harmful use |
| F102 | F10.2 Dependence syndrome |
| F103 | F10.3 Withdrawal state |
| F104 | F10.4 Withdrawal state with delirium |
| F105 | F10.5 Psychotic disorder |
| F106 | F10.6 Amnesic syndrome |
| F107 | F10.7 Residual and late-onset psychotic disorder |
| F108 | F10.8 Other mental and behavioural disorders |
| F109 | F10.9 Unspecified mental and behavioural disorder |
| F11 | F11 Mental and behavioural disorders due to use of opioids |
| F110 | F11.0 Acute intoxication |
| F111 | F11.1 Harmful use |
| F112 | F11.2 Dependence syndrome |
| F113 | F11.3 Withdrawal state |
| F114 | F11.4 Withdrawal state with delirium |
| F115 | F11.5 Psychotic disorder |
| F116 | F11.6 Amnesic syndrome |
| F117 | F11.7 Residual and late-onset psychotic disorder |
| F118 | F11.8 Other mental and behavioural disorders |
| F119 | F11.9 Unspecified mental and behavioural disorder |
| F12 | F12 Mental and behavioural disorders due to use of cannabinoids |
| F120 | F12.0 Acute intoxication |
| F121 | F12.1 Harmful use |
| F122 | F12.2 Dependence syndrome |
| F123 | F12.3 Withdrawal state |
| F124 | F12.4 Withdrawal state with delirium |
| F125 | F12.5 Psychotic disorder |
| F126 | F12.6 Amnesic syndrome |
| F127 | F12.7 Residual and late-onset psychotic disorder |
| F128 | F12.8 Other mental and behavioural disorders |
| F129 | F12.9 Unspecified mental and behavioural disorder |
| F13 | F13 Mental and behavioural disorders due to use of sedatives or hypnotics |
| F130 | F13.0 Acute intoxication |
| F131 | F13.1 Harmful use |
| F132 | F13.2 Dependence syndrome |
| F133 | F13.3 Withdrawal state |
| F134 | F13.4 Withdrawal state with delirium |
| F135 | F13.5 Psychotic disorder |
| F136 | F13.6 Amnesic syndrome |
| F137 | F13.7 Residual and late-onset psychotic disorder |
| F138 | F13.8 Other mental and behavioural disorders |
| F139 | F13.9 Unspecified mental and behavioural disorder |
| F14 | F14 Mental and behavioural disorders due to use of cocaine |
| F140 | F14.0 Acute intoxication |
| F141 | F14.1 Harmful use |
| F142 | F14.2 Dependence syndrome |
| F143 | F14.3 Withdrawal state |
| F144 | F14.4 Withdrawal state with delirium |
| F145 | F14.5 Psychotic disorder |
| F146 | F14.6 Amnesic syndrome |
| F147 | F14.7 Residual and late-onset psychotic disorder |
| F148 | F14.8 Other mental and behavioural disorders |
| F149 | F14.9 Unspecified mental and behavioural disorder |
| F15 | F15 Mental and behavioural disorders due to use of other stimulants |
| F150 | F15.0 Acute intoxication |
| F151 | F15.1 Harmful use |
| F152 | F15.2 Dependence syndrome |
| F153 | F15.3 Withdrawal state |
| F154 | F15.4 Withdrawal state with delirium |
| F155 | F15.5 Psychotic disorder |
| F156 | F15.6 Amnesic syndrome |
| F157 | F15.7 Residual and late-onset psychotic disorder |
| F158 | F15.8 Other mental and behavioural disorders |
| F159 | F15.9 Unspecified mental and behavioural disorder |
| F16 | F16 Mental and behavioural disorders due to use of hallucinogens |
| F160 | F16.0 Acute intoxication |
| F161 | F16.1 Harmful use |
| F162 | F16.2 Dependence syndrome |
| F163 | F16.3 Withdrawal state |
| F164 | F16.4 Withdrawal state with delirium |
| F165 | F16.5 Psychotic disorder |
| F166 | F16.6 Amnesic syndrome |
| F167 | F16.7 Residual and late-onset psychotic disorder |
| F168 | F16.8 Other mental and behavioural disorders |
| F169 | F16.9 Unspecified mental and behavioural disorder |
| F17 | F17 Mental and behavioural disorders due to use of tobacco |
| F170 | F17.0 Acute intoxication |
| F171 | F17.1 Harmful use |
| F172 | F17.2 Dependence syndrome |
| F173 | F17.3 Withdrawal state |
| F174 | F17.4 Withdrawal state with delirium |
| F175 | F17.5 Psychotic disorder |
| F176 | F17.6 Amnesic syndrome |
| F177 | F17.7 Residual and late-onset psychotic disorder |
| F178 | F17.8 Other mental and behavioural disorders |
| F179 | F17.9 Unspecified mental and behavioural disorder |
| F18 | F18 Mental and behavioural disorders due to use of volatile solvents |
| F180 | F18.0 Acute intoxication |
| F181 | F18.1 Harmful use |
| F182 | F18.2 Dependence syndrome |
| F183 | F18.3 Withdrawal state |
| F184 | F18.4 Withdrawal state with delirium |
| F185 | F18.5 Psychotic disorder |
| F186 | F18.6 Amnesic syndrome |
| F187 | F18.7 Residual and late-onset psychotic disorder |
| F188 | F18.8 Other mental and behavioural disorders |
| F189 | F18.9 Unspecified mental and behavioural disorder |
| F19 | F19 Mental and behavioural disorders due to multiple drug use and use of other psychoactive substances |
| F190 | F19.0 Acute intoxication |
| F191 | F19.1 Harmful use |
| F192 | F19.2 Dependence syndrome |
| F193 | F19.3 Withdrawal state |
| F194 | F19.4 Withdrawal state with delirium |
| F195 | F19.5 Psychotic disorder |
| F196 | F19.6 Amnesic syndrome |
| F197 | F19.7 Residual and late-onset psychotic disorder |
| F198 | F19.8 Other mental and behavioural disorders |
| F199 | F19.9 Unspecified mental and behavioural disorder |
| F20 | F20 Schizophrenia |
| F200 | F20.0 Paranoid schizophrenia |
| F201 | F20.1 Hebephrenic schizophrenia |
| F202 | F20.2 Catatonic schizophrenia |
| F203 | F20.3 Undifferentiated schizophrenia |
| F204 | F20.4 Postschizophrenic depression |
| F205 | F20.5 Residual schizophrenia |
| F206 | F20.6 Simple schizophrenia |
| F208 | F20.8 Other schizophrenia |
| F209 | F20.9 Schizophrenia |
| F21 | F21 Schizotypal disorder |
| F22 | F22 Persistent delusional disorders |
| F220 | F22.0 Delusional disorder |
| F228 | F22.8 Other persistent delusional disorders |
| F229 | F22.9 Persistent delusional disorder |
| F23 | F23 Acute and transient psychotic disorders |
| F230 | F23.0 Acute polymorphic psychotic disorder without symptoms of schizophrenia |
| F231 | F23.1 Acute polymorphic psychotic disorder with symptoms of schizophrenia |
| F232 | F23.2 Acute schizophrenia-like psychotic disorder |
| F233 | F23.3 Other acute predominantly delusional psychotic disorders |
| F238 | F23.8 Other acute and transient psychotic disorders |
| F239 | F23.9 Acute and transient psychotic disorder |
| F24 | F24 Induced delusional disorder |
| F25 | F25 Schizoaffective disorders |
| F250 | F25.0 Schizoaffective disorder |
| F251 | F25.1 Schizoaffective disorder |
| F252 | F25.2 Schizoaffective disorder |
| F258 | F25.8 Other schizoaffective disorders |
| F259 | F25.9 Schizoaffective disorder |
| F28 | F28 Other nonorganic psychotic disorders |
| F29 | F29 Unspecified nonorganic psychosis |
| F30 | F30 Manic episode |
| F300 | F30.0 Hypomania |
| F301 | F30.1 Mania without psychotic symptoms |
| F302 | F30.2 Mania with psychotic symptoms |
| F308 | F30.8 Other manic episodes |
| F309 | F30.9 Manic episode |
| F31 | F31 Bipolar affective disorder |
| F310 | F31.0 Bipolar affective disorder |
| F311 | F31.1 Bipolar affective disorder |
| F312 | F31.2 Bipolar affective disorder |
| F313 | F31.3 Bipolar affective disorder |
| F314 | F31.4 Bipolar affective disorder |
| F315 | F31.5 Bipolar affective disorder |
| F316 | F31.6 Bipolar affective disorder |
| F317 | F31.7 Bipolar affective disorder |
| F318 | F31.8 Other bipolar affective disorders |
| F319 | F31.9 Bipolar affective disorder |
| F32 | F32 Depressive episode |
| F320 | F32.0 Mild depressive episode |
| F321 | F32.1 Moderate depressive episode |
| F322 | F32.2 Severe depressive episode without psychotic symptoms |
| F323 | F32.3 Severe depressive episode with psychotic symptoms |
| F328 | F32.8 Other depressive episodes |
| F329 | F32.9 Depressive episode |
| F33 | F33 Recurrent depressive disorder |
| F330 | F33.0 Recurrent depressive disorder |
| F331 | F33.1 Recurrent depressive disorder |
| F332 | F33.2 Recurrent depressive disorder |
| F333 | F33.3 Recurrent depressive disorder |
| F334 | F33.4 Recurrent depressive disorder |
| F338 | F33.8 Other recurrent depressive disorders |
| F339 | F33.9 Recurrent depressive disorder |
| F34 | F34 Persistent mood [affective] disorders |
| F340 | F34.0 Cyclothymia |
| F341 | F34.1 Dysthymia |
| F348 | F34.8 Other persistent mood [affective] disorders |
| F349 | F34.9 Persistent mood [affective] disorder |
| F38 | F38 Other mood [affective] disorders |
| F380 | F38.0 Other single mood [affective] disorders |
| F381 | F38.1 Other recurrent mood [affective] disorders |
| F388 | F38.8 Other specified mood [affective] disorders |
| F39 | F39 Unspecified mood [affective] disorder |
| F40 | F40 Phobic anxiety disorders |
| F400 | F40.0 Agoraphobia |
| F401 | F40.1 Social phobias |
| F402 | F40.2 Specific (isolated) phobias |
| F408 | F40.8 Other phobic anxiety disorders |
| F409 | F40.9 Phobic anxiety disorder |
| F41 | F41 Other anxiety disorders |
| F410 | F41.0 Panic disorder [episodic paroxysmal anxiety] |
| F411 | F41.1 Generalised anxiety disorder |
| F412 | F41.2 Mixed anxiety and depressive disorder |
| F413 | F41.3 Other mixed anxiety disorders |
| F418 | F41.8 Other specified anxiety disorders |
| F419 | F41.9 Anxiety disorder |
| F42 | F42 Obsessive-compulsive disorder |
| F420 | F42.0 Predominantly obsessional thoughts or ruminations |
| F421 | F42.1 Predominantly compulsive acts [obsessional rituals] |
| F422 | F42.2 Mixed obsessional thoughts and acts |
| F428 | F42.8 Other obsessive-compulsive disorders |
| F429 | F42.9 Obsessive-compulsive disorder |
| F43 | F43 Reaction to severe stress |
| F430 | F43.0 Acute stress reaction |
| F431 | F43.1 Posttraumatic stress disorder |
| F432 | F43.2 Adjustment disorders |
| F438 | F43.8 Other reactions to severe stress |
| F439 | F43.9 Reaction to severe stress |
| F44 | F44 Dissociative [conversion] disorders |
| F440 | F44.0 Dissociative amnesia |
| F441 | F44.1 Dissociative fugue |
| F442 | F44.2 Dissociative stupor |
| F443 | F44.3 Trance and possession disorders |
| F444 | F44.4 Dissociative motor disorders |
| F445 | F44.5 Dissociative convulsions |
| F446 | F44.6 Dissociative anaesthesia and sensory loss |
| F447 | F44.7 Mixed dissociative [conversion] disorders |
| F448 | F44.8 Other dissociative [conversion] disorders |
| F449 | F44.9 Dissociative [conversion] disorder |
| F45 | F45 Somatoform disorders |
| F450 | F45.0 Somatisation disorder |
| F451 | F45.1 Undifferentiated somatoform disorder |
| F452 | F45.2 Hypochondriacal disorder |
| F453 | F45.3 Somatoform autonomic dysfunction |
| F454 | F45.4 Persistent somatoform pain disorder |
| F458 | F45.8 Other somatoform disorders |
| F459 | F45.9 Somatoform disorder |
| F48 | F48 Other neurotic disorders |
| F480 | F48.0 Neurasthenia |
| F481 | F48.1 Depersonalisation-derealisation syndrome |
| F488 | F48.8 Other specified neurotic disorders |
| F489 | F48.9 Neurotic disorder |
| F50 | F50 Eating disorders |
| F500 | F50.0 Anorexia nervosa |
| F501 | F50.1 Atypical anorexia nervosa |
| F502 | F50.2 Bulimia nervosa |
| F503 | F50.3 Atypical bulimia nervosa |
| F504 | F50.4 Overeating associated with other psychological disturbances |
| F505 | F50.5 Vomiting associated with other psychological disturbances |
| F508 | F50.8 Other eating disorders |
| F509 | F50.9 Eating disorder |
| F51 | F51 Nonorganic sleep disorders |
| F510 | F51.0 Nonorganic insomnia |
| F511 | F51.1 Nonorganic hypersomnia |
| F512 | F51.2 Nonorganic disorder of the sleep-wake schedule |
| F513 | F51.3 Sleepwalking [somnambulism] |
| F514 | F51.4 Sleep terrors [night terrors] |
| F515 | F51.5 Nightmares |
| F518 | F51.8 Other nonorganic sleep disorders |
| F519 | F51.9 Nonorganic sleep disorder |
| F52 | F52 Sexual dysfunction |
| F520 | F52.0 Lack or loss of sexual desire |
| F521 | F52.1 Sexual aversion and lack of sexual enjoyment |
| F522 | F52.2 Failure of genital response |
| F523 | F52.3 Orgasmic dysfunction |
| F524 | F52.4 Premature ejaculation |
| F525 | F52.5 Nonorganic vaginismus |
| F526 | F52.6 Nonorganic dyspareunia |
| F527 | F52.7 Excessive sexual drive |
| F528 | F52.8 Other sexual dysfunction |
| F529 | F52.9 Unspecified sexual dysfunction |
| F53 | F53 Mental and behavioural disorders associated with the puerperium |
| F530 | F53.0 Mild mental and behavioural disorders associated with the puerperium |
| F531 | F53.1 Severe mental and behavioural disorders associated with the puerperium |
| F538 | F53.8 Other mental and behavioural disorders associated with the puerperium |
| F539 | F53.9 Puerperal mental disorder |
| F54 | F54 Psychological and behavioural factors associated with disorders or diseases classified elsewhere |
| F55 | F55 Abuse of non-dependence-producing substances |
| F59 | F59 Unspecified behavioural syndromes associated with physiological disturbances and physical factors |
| F60 | F60 Specific personality disorders |
| F600 | F60.0 Paranoid personality disorder |
| F601 | F60.1 Schizoid personality disorder |
| F602 | F60.2 Dissocial personality disorder |
| F603 | F60.3 Emotionally unstable personality disorder |
| F604 | F60.4 Histrionic personality disorder |
| F605 | F60.5 Anankastic personality disorder |
| F606 | F60.6 Anxious [avoidant] personality disorder |
| F607 | F60.7 Dependent personality disorder |
| F608 | F60.8 Other specific personality disorders |
| F609 | F60.9 Personality disorder |
| F61 | F61 Mixed and other personality disorders |
| F62 | F62 Enduring personality changes |
| F620 | F62.0 Enduring personality change after catastrophic experience |
| F621 | F62.1 Enduring personality change after psychiatric illness |
| F628 | F62.8 Other enduring personality changes |
| F629 | F62.9 Enduring personality change |
| F63 | F63 Habit and impulse disorders |
| F630 | F63.0 Pathological gambling |
| F631 | F63.1 Pathological fire-setting [pyromania] |
| F632 | F63.2 Pathological stealing [kleptomania] |
| F633 | F63.3 Trichotillomania |
| F638 | F63.8 Other habit and impulse disorders |
| F639 | F63.9 Habit and impulse disorder |
| F64 | F64 Gender identity disorders |
| F640 | F64.0 Transsexualism |
| F641 | F64.1 Dual-role transvestism |
| F642 | F64.2 Gender identity disorder of childhood |
| F648 | F64.8 Other gender identity disorders |
| F649 | F64.9 Gender identity disorder |
| F65 | F65 Disorders of sexual preference |
| F650 | F65.0 Fetishism |
| F651 | F65.1 Fetishistic transvestism |
| F652 | F65.2 Exhibitionism |
| F653 | F65.3 Voyeurism |
| F654 | F65.4 Paedophilia |
| F655 | F65.5 Sadomasochism |
| F656 | F65.6 Multiple disorders of sexual preference |
| F658 | F65.8 Other disorders of sexual preference |
| F659 | F65.9 Disorder of sexual preference |
| F66 | F66 Psychological and behavioural disorders associated with sexual development and orientation |
| F660 | F66.0 Sexual maturation disorder |
| F661 | F66.1 Egodystonic sexual orientation |
| F662 | F66.2 Sexual relationship disorder |
| F668 | F66.8 Other psychosexual development disorders |
| F669 | F66.9 Psychosexual development disorder |
| F68 | F68 Other disorders of adult personality and behaviour |
| F680 | F68.0 Elaboration of physical symptoms for psychological reasons |
| F681 | F68.1 Intentional production or feigning of symptoms or disabilities |
| F688 | F68.8 Other specified disorders of adult personality and behaviour |
| F69 | F69 Unspecified disorder of adult personality and behaviour |
| F70 | F70 Mild mental retardation |
| F700 | F70.0 Mild mental retardation (With the statement of no |
| F701 | F70.1 Mild mental retardation (Significant impairment of behaviour requiring attention or treatment) |
| F708 | F70.8 Mild mental retardation (Other impairments of behaviour) |
| F709 | F70.9 Mild mental retardation (Without mention of impairment of behaviour) |
| F71 | F71 Moderate mental retardation |
| F710 | F71.0 Moderate mental retardation (With the statement of no |
| F711 | F71.1 Moderate mental retardation (Significant impairment of behaviour requiring attention or treatment) |
| F718 | F71.8 Moderate mental retardation (Other impairments of behaviour) |
| F719 | F71.9 Moderate mental retardation (Without mention of impairment of behaviour) |
| F72 | F72 Severe mental retardation |
| F720 | F72.0 Severe mental retardation (With the statement of no |
| F721 | F72.1 Severe mental retardation (Significant impairment of behaviour requiring attention or treatment) |
| F728 | F72.8 Severe mental retardation (Other impairments of behaviour) |
| F729 | F72.9 Severe mental retardation (Without mention of impairment of behaviour) |
| F73 | F73 Profound mental retardation |
| F730 | F73.0 Profound mental retardation (With the statement of no |
| F731 | F73.1 Profound mental retardation (Significant impairment of behaviour requiring attention or treatment) |
| F738 | F73.8 Profound mental retardation (Other impairments of behaviour) |
| F739 | F73.9 Profound mental retardation (Without mention of impairment of behaviour) |
| F78 | F78 Other mental retardation |
| F780 | F78.0 Other mental retardation (With the statement of no |
| F781 | F78.1 Other mental retardation (Significant impairment of behaviour requiring attention or treatment) |
| F788 | F78.8 Other mental retardation (Other impairments of behaviour) |
| F789 | F78.9 Other mental retardation (Without mention of impairment of behaviour) |
| F79 | F79 Unspecified mental retardation |
| F790 | F79.0 Unspecified mental retardation (With the statement of no |
| F791 | F79.1 Unspecified mental retardation (Significant impairment of behaviour requiring attention or treatment) |
| F798 | F79.8 Unspecified mental retardation (Other impairments of behaviour) |
| F799 | F79.9 Unspecified mental retardation (Without mention of impairment of behaviour) |
| F80 | F80 Specific developmental disorders of speech and language |
| F800 | F80.0 Specific speech articulation disorder |
| F801 | F80.1 Expressive language disorder |
| F802 | F80.2 Receptive language disorder |
| F803 | F80.3 Acquired aphasia with epilepsy [Landau-Kleffner] |
| F808 | F80.8 Other developmental disorders of speech and language |
| F809 | F80.9 Developmental disorder of speech and language |
| F81 | F81 Specific developmental disorders of scholastic skills |
| F810 | F81.0 Specific reading disorder |
| F811 | F81.1 Specific spelling disorder |
| F812 | F81.2 Specific disorder of arithmetical skills |
| F813 | F81.3 Mixed disorder of scholastic skills |
| F818 | F81.8 Other developmental disorders of scholastic skills |
| F819 | F81.9 Developmental disorder of scholastic skills |
| F82 | F82 Specific developmental disorder of motor function |
| F83 | F83 Mixed specific developmental disorders |
| F84 | F84 Pervasive developmental disorders |
| F840 | F84.0 Childhood autism |
| F841 | F84.1 Atypical autism |
| F842 | F84.2 Rett's syndrome |
| F843 | F84.3 Other childhood disintegrative disorder |
| F844 | F84.4 Overactive disorder associated with mental retardation and stereotyped movements |
| F845 | F84.5 Asperger's syndrome |
| F848 | F84.8 Other pervasive developmental disorders |
| F849 | F84.9 Pervasive developmental disorder |
| F88 | F88 Other disorders of psychological development |
| F89 | F89 Unspecified disorder of psychological development |
| F90 | F90 Hyperkinetic disorders |
| F900 | F90.0 Disturbance of activity and attention |
| F901 | F90.1 Hyperkinetic conduct disorder |
| F908 | F90.8 Other hyperkinetic disorders |
| F909 | F90.9 Hyperkinetic disorder |
| F91 | F91 Conduct disorders |
| F910 | F91.0 Conduct disorder confined to the family context |
| F911 | F91.1 Unsocialised conduct disorder |
| F912 | F91.2 Socialised conduct disorder |
| F913 | F91.3 Oppositional defiant disorder |
| F918 | F91.8 Other conduct disorders |
| F919 | F91.9 Conduct disorder |
| F92 | F92 Mixed disorders of conduct and emotions |
| F920 | F92.0 Depressive conduct disorder |
| F928 | F92.8 Other mixed disorders of conduct and emotions |
| F929 | F92.9 Mixed disorder of conduct and emotions |
| F93 | F93 Emotional disorders with onset specific to childhood |
| F930 | F93.0 Separation anxiety disorder of childhood |
| F931 | F93.1 Phobic anxiety disorder of childhood |
| F932 | F93.2 Social anxiety disorder of childhood |
| F933 | F93.3 Sibling rivalry disorder |
| F938 | F93.8 Other childhood emotional disorders |
| F939 | F93.9 Childhood emotional disorder |
| F94 | F94 Disorders of social functioning with onset specific to childhood and adolescence |
| F940 | F94.0 Elective mutism |
| F941 | F94.1 Reactive attachment disorder of childhood |
| F942 | F94.2 Disinhibited attachment disorder of childhood |
| F948 | F94.8 Other childhood disorders of social functioning |
| F949 | F94.9 Childhood disorder of social functioning |
| F95 | F95 Tic disorders |
| F950 | F95.0 Transient tic disorder |
| F951 | F95.1 Chronic motor or vocal tic disorder |
| F952 | F95.2 Combined vocal and multiple motor tic disorder [de la Tourette] |
| F958 | F95.8 Other tic disorders |
| F959 | F95.9 Tic disorder |
| F98 | F98 Other behavioural and emotional disorders with onset usually occurring in childhood and adolescence |
| F980 | F98.0 Nonorganic enuresis |
| F981 | F98.1 Nonorganic encopresis |
| F982 | F98.2 Feeding disorder of infancy and childhood |
| F983 | F98.3 Pica of infancy and childhood |
| F984 | F98.4 Stereotyped movement disorders |
| F985 | F98.5 Stuttering [stammering] |
| F986 | F98.6 Cluttering |
| F988 | F98.8 Other specified behavioural and emotional disorders with onset usually occurring in childhood and adolescence |
| F989 | F98.9 Unspecified behavioural and emotional disorders with onset usually occurring in childhood and adolescence |
| F99 | F99 Mental disorder |
| G00 | G00 Bacterial meningitis |
| G000 | G00.0 Haemophilus meningitis |
| G001 | G00.1 Pneumococcal meningitis |
| G002 | G00.2 Streptococcal meningitis |
| G003 | G00.3 Staphylococcal meningitis |
| G008 | G00.8 Other bacterial meningitis |
| G009 | G00.9 Bacterial meningitis |
| G01 | G01 Meningitis in bacterial diseases classified elsewhere |
| G02 | G02 Meningitis in other infectious and parasitic diseases classified elsewhere |
| G020 | G02.0 Meningitis in viral diseases classified elsewhere |
| G021 | G02.1 Meningitis in mycoses |
| G028 | G02.8 Meningitis in other specified infectious and parasitic diseases classified elsewhere |
| G03 | G03 Meningitis due to other and unspecified causes |
| G030 | G03.0 Nonpyogenic meningitis |
| G031 | G03.1 Chronic meningitis |
| G032 | G03.2 Benign recurrent meningitis [Mollaret] |
| G038 | G03.8 Meningitis due to other specified causes |
| G039 | G03.9 Meningitis |
| G04 | G04 Encephalitis |
| G040 | G04.0 Acute disseminated encephalitis |
| G041 | G04.1 Tropical spastic paraplegia |
| G042 | G04.2 Bacterial meningoencephalitis and meningomyelitis |
| G048 | G04.8 Other encephalitis |
| G049 | G04.9 Encephalitis |
| G05 | G05 Encephalitis |
| G050 | G05.0 Encephalitis |
| G051 | G05.1 Encephalitis |
| G052 | G05.2 Encephalitis |
| G058 | G05.8 Encephalitis |
| G06 | G06 Intracranial and intraspinal abscess and granuloma |
| G060 | G06.0 Intracranial abscess and granuloma |
| G061 | G06.1 Intraspinal abscess and granuloma |
| G062 | G06.2 Extradural and subdural abscess |
| G07 | G07 Intracranial and intraspinal abscess and granuloma in diseases classified elsewhere |
| G08 | G08 Intracranial and intraspinal phlebitis and thrombophlebitis |
| G09 | G09 Sequelae of inflammatory diseases of central nervous system |
| G10 | G10 Huntington's disease |
| G11 | G11 Hereditary ataxia |
| G110 | G11.0 Congenital nonprogressive ataxia |
| G111 | G11.1 Early-onset cerebellar ataxia |
| G112 | G11.2 Late-onset cerebellar ataxia |
| G113 | G11.3 Cerebellar ataxia with defective DNA repair |
| G114 | G11.4 Hereditary spastic paraplegia |
| G118 | G11.8 Other hereditary ataxias |
| G119 | G11.9 Hereditary ataxia |
| G12 | G12 Spinal muscular atrophy and related syndromes |
| G120 | G12.0 Infantile spinal muscular atrophy |
| G121 | G12.1 Other inherited spinal muscular atrophy |
| G122 | G12.2 Motor neuron disease |
| G128 | G12.8 Other spinal muscular atrophies and related syndromes |
| G129 | G12.9 Spinal muscular atrophy |
| G13 | G13 Systemic atrophies primarily affecting central nervous system in diseases classified elsewhere |
| G130 | G13.0 Paraneoplastic neuromyopathy and neuropathy |
| G131 | G13.1 Other systemic atrophy primarily affecting central nervous system in neoplastic disease |
| G132 | G13.2 Systemic atrophy primarily affecting central nervous system in myxoedema |
| G138 | G13.8 Systemic atrophy primarily affecting central nervous system in other diseases classified elsewhere |
| G14 | G14 Postpolio syndrome |
| G20 | G20 Parkinson's disease |
| G21 | G21 Secondary Parkinsonism |
| G210 | G21.0 Malignant neuroleptic syndrome |
| G211 | G21.1 Other drug-induced secondary Parkinsonism |
| G212 | G21.2 Secondary Parkinsonism due to other external agents |
| G213 | G21.3 Postencephalitic Parkinsonism |
| G214 | G21.4 Vascular parkinsonism |
| G218 | G21.8 Other secondary Parkinsonism |
| G219 | G21.9 Secondary Parkinsonism |
| G22 | G22 Parkinsonism in diseases classified elsewhere |
| G23 | G23 Other degenerative diseases of basal ganglia |
| G230 | G23.0 Hallervorden-Spatz disease |
| G231 | G23.1 Progressive supranuclear ophthalmoplegia [Steele-Richardson-Olszewski] |
| G232 | G23.2 Striatonigral degeneration |
| G233 | G23.3 Multiple system atrophy |
| G238 | G23.8 Other specified degenerative diseases of basal ganglia |
| G239 | G23.9 Degenerative disease of basal ganglia |
| G24 | G24 Dystonia |
| G240 | G24.0 Drug-induced dystonia |
| G241 | G24.1 Idiopathic familial dystonia |
| G242 | G24.2 Idiopathic nonfamilial dystonia |
| G243 | G24.3 Spasmodic torticollis |
| G244 | G24.4 Idiopathic orofacial dystonia |
| G245 | G24.5 Blepharospasm |
| G248 | G24.8 Other dystonia |
| G249 | G24.9 Dystonia |
| G25 | G25 Other extrapyramidal and movement disorders |
| G250 | G25.0 Essential tremor |
| G251 | G25.1 Drug-induced tremor |
| G252 | G25.2 Other specified forms of tremor |
| G253 | G25.3 Myoclonus |
| G254 | G25.4 Drug-induced chorea |
| G255 | G25.5 Other chorea |
| G256 | G25.6 Drug-induced tics and other tics of organic origin |
| G258 | G25.8 Other specified extrapyramidal and movement disorders |
| G259 | G25.9 Extrapyramidal and movement disorder |
| G26 | G26 Extrapyramidal and movement disorders in diseases classified elsewhere |
| G30 | G30 Alzheimer's disease |
| G300 | G30.0 Alzheimer's disease with early onset |
| G301 | G30.1 Alzheimer's disease with late onset |
| G308 | G30.8 Other Alzheimer's disease |
| G309 | G30.9 Alzheimer's disease |
| G31 | G31 Other degenerative diseases of nervous system |
| G310 | G31.0 Circumscribed brain atrophy |
| G311 | G31.1 Senile degeneration of brain |
| G312 | G31.2 Degeneration of nervous system due to alcohol |
| G318 | G31.8 Other specified degenerative diseases of nervous system |
| G319 | G31.9 Degenerative disease of nervous system |
| G32 | G32 Other degenerative disorders of nervous system in diseases classified elsewhere |
| G320 | G32.0 Subacute combined degeneration of spinal cord in diseases classified elsewhere |
| G328 | G32.8 Other specified degenerative disorders of nervous system in diseases classified elsewhere |
| G35 | G35 Multiple sclerosis |
| G36 | G36 Other acute disseminated demyelination |
| G360 | G36.0 Neuromyelitis optica [Devic] |
| G361 | G36.1 Acute and subacute haemorrhagic leukoencephalitis [Hurst] |
| G368 | G36.8 Other specified acute disseminated demyelination |
| G369 | G36.9 Acute disseminated demyelination |
| G37 | G37 Other demyelinating diseases of central nervous system |
| G370 | G37.0 Diffuse sclerosis |
| G371 | G37.1 Central demyelination of corpus callosum |
| G372 | G37.2 Central pontine myelinolysis |
| G373 | G37.3 Acute transverse myelitis in demyelinating disease of central nervous system |
| G374 | G37.4 Subacute necrotising myelitis |
| G375 | G37.5 Concentric sclerosis [Balo] |
| G378 | G37.8 Other specified demyelinating diseases of central nervous system |
| G379 | G37.9 Demyelinating disease of central nervous system |
| G40 | G40 Epilepsy |
| G400 | G40.0 Localisation-related (focal) (partial) idiopathic epilepsy and epileptic syndromes with seizures of localised onset |
| G401 | G40.1 Localisation-related (focal) (partial) symptomatic epilepsy and epileptic syndromes with simple partial seizures |
| G402 | G40.2 Localisation-related (focal) (partial) symptomatic epilepsy and epileptic syndromes with complex partial seizures |
| G403 | G40.3 Generalised idiopathic epilepsy and epileptic syndromes |
| G404 | G40.4 Other generalised epilepsy and epileptic syndromes |
| G405 | G40.5 Special epileptic syndromes |
| G406 | G40.6 Grand mal seizures |
| G407 | G40.7 Petit mal |
| G408 | G40.8 Other epilepsy |
| G409 | G40.9 Epilepsy |
| G41 | G41 Status epilepticus |
| G410 | G41.0 Grand mal status epilepticus |
| G411 | G41.1 Petit mal status epilepticus |
| G412 | G41.2 Complex partial status epilepticus |
| G418 | G41.8 Other status epilepticus |
| G419 | G41.9 Status epilepticus |
| G43 | G43 Migraine |
| G430 | G43.0 Migraine without aura [common migraine] |
| G431 | G43.1 Migraine with aura [classical migraine] |
| G432 | G43.2 Status migrainosus |
| G433 | G43.3 Complicated migraine |
| G438 | G43.8 Other migraine |
| G439 | G43.9 Migraine |
| G44 | G44 Other headache syndromes |
| G440 | G44.0 Cluster headache syndrome |
| G441 | G44.1 Vascular headache |
| G442 | G44.2 Tension-type headache |
| G443 | G44.3 Chronic posttraumatic headache |
| G444 | G44.4 Drug-induced headache |
| G448 | G44.8 Other specified headache syndromes |
| G45 | G45 Transient cerebral ischaemic attacks and related syndromes |
| G450 | G45.0 Vertebro-basilar artery syndrome |
| G451 | G45.1 Carotid artery syndrome (hemispheric) |
| G452 | G45.2 Multiple and bilateral precerebral artery syndromes |
| G453 | G45.3 Amaurosis fugax |
| G454 | G45.4 Transient global amnesia |
| G458 | G45.8 Other transient cerebral ischaemic attacks and related syndromes |
| G459 | G45.9 Transient cerebral ischaemic attack |
| G46 | G46 Vascular syndromes of brain in cerebrovascular diseases |
| G460 | G46.0 Middle cerebral artery syndrome |
| G461 | G46.1 Anterior cerebral artery syndrome |
| G462 | G46.2 Posterior cerebral artery syndrome |
| G463 | G46.3 Brain stem stroke syndrome |
| G464 | G46.4 Cerebellar stroke syndrome |
| G465 | G46.5 Pure motor lacunar syndrome |
| G466 | G46.6 Pure sensory lacunar syndrome |
| G467 | G46.7 Other lacunar syndromes |
| G468 | G46.8 Other vascular syndromes of brain in cerebrovascular diseases |
| G47 | G47 Sleep disorders |
| G470 | G47.0 Disorders of initiating and maintaining sleep [insomnias] |
| G471 | G47.1 Disorders of excessive somnolence [hypersomnias] |
| G472 | G47.2 Disorders of the sleep-wake schedule |
| G473 | G47.3 Sleep apnoea |
| G474 | G47.4 Narcolepsy and cataplexy |
| G478 | G47.8 Other sleep disorders |
| G479 | G47.9 Sleep disorder |
| G50 | G50 Disorders of trigeminal nerve |
| G500 | G50.0 Trigeminal neuralgia |
| G501 | G50.1 Atypical facial pain |
| G508 | G50.8 Other disorders of trigeminal nerve |
| G509 | G50.9 Disorder of trigeminal nerve |
| G51 | G51 Facial nerve disorders |
| G510 | G51.0 Bell's palsy |
| G511 | G51.1 Geniculate ganglionitis |
| G512 | G51.2 Melkersson's syndrome |
| G513 | G51.3 Clonic hemifacial spasm |
| G514 | G51.4 Facial myokymia |
| G518 | G51.8 Other disorders of facial nerve |
| G519 | G51.9 Disorder of facial nerve |
| G52 | G52 Disorders of other cranial nerves |
| G520 | G52.0 Disorders of olfactory nerve |
| G521 | G52.1 Disorders of glossopharyngeal nerve |
| G522 | G52.2 Disorders of vagus nerve |
| G523 | G52.3 Disorders of hypoglossal nerve |
| G527 | G52.7 Disorders of multiple cranial nerves |
| G528 | G52.8 Disorders of other specified cranial nerves |
| G529 | G52.9 Cranial nerve disorder |
| G53 | G53 Cranial nerve disorders in diseases classified elsewhere |
| G530 | G53.0 Postzoster neuralgia |
| G531 | G53.1 Multiple cranial nerve palsies in infectious and parasitic diseases classified elsewhere |
| G532 | G53.2 Multiple cranial nerve palsies in sarcoidosis |
| G533 | G53.3 Multiple cranial nerve palsies in neoplastic disease |
| G538 | G53.8 Other cranial nerve disorders in other diseases classified elsewhere |
| G54 | G54 Nerve root and plexus disorders |
| G540 | G54.0 Brachial plexus disorders |
| G541 | G54.1 Lumbosacral plexus disorders |
| G542 | G54.2 Cervical root disorders |
| G543 | G54.3 Thoracic root disorders |
| G544 | G54.4 Lumbosacral root disorders |
| G545 | G54.5 Neuralgic amyotrophy |
| G546 | G54.6 Phantom limb syndrome with pain |
| G547 | G54.7 Phantom limb syndrome without pain |
| G548 | G54.8 Other nerve root and plexus disorders |
| G549 | G54.9 Nerve root and plexus disorder |
| G55 | G55 Nerve root and plexus compressions in diseases classified elsewhere |
| G550 | G55.0 Nerve root and plexus compressions in neoplastic disease |
| G551 | G55.1 Nerve root and plexus compressions in intervertebral disk disorders |
| G552 | G55.2 Nerve root and plexus compressions in spondylosis |
| G553 | G55.3 Nerve root and plexus compressions in other dorsopathies |
| G558 | G55.8 Nerve root and plexus compressions in other diseases classified elsewhere |
| G56 | G56 Mononeuropathies of upper limb |
| G560 | G56.0 Carpal tunnel syndrome |
| G561 | G56.1 Other lesions of median nerve |
| G562 | G56.2 Lesion of ulnar nerve |
| G563 | G56.3 Lesion of radial nerve |
| G564 | G56.4 Causalgia |
| G568 | G56.8 Other mononeuropathies of upper limb |
| G569 | G56.9 Mononeuropathy of upper limb |
| G57 | G57 Mononeuropathies of lower limb |
| G570 | G57.0 Lesion of sciatic nerve |
| G571 | G57.1 Meralgia paraesthetica |
| G572 | G57.2 Lesion of femoral nerve |
| G573 | G57.3 Lesion of lateral popliteal nerve |
| G574 | G57.4 Lesion of medial popliteal nerve |
| G575 | G57.5 Tarsal tunnel syndrome |
| G576 | G57.6 Lesion of plantar nerve |
| G578 | G57.8 Other mononeuropathies of lower limb |
| G579 | G57.9 Mononeuropathy of lower limb |
| G58 | G58 Other mononeuropathies |
| G580 | G58.0 Intercostal neuropathy |
| G587 | G58.7 Mononeuritis multiplex |
| G588 | G58.8 Other specified mononeuropathies |
| G589 | G58.9 Mononeuropathy |
| G59 | G59 Mononeuropathy in diseases classified elsewhere |
| G590 | G59.0 Diabetic mononeuropathy |
| G598 | G59.8 Other mononeuropathies in diseases classified elsewhere |
| G60 | G60 Hereditary and idiopathic neuropathy |
| G600 | G60.0 Hereditary motor and sensory neuropathy |
| G601 | G60.1 Refsum's disease |
| G602 | G60.2 Neuropathy in association with hereditary ataxia |
| G603 | G60.3 Idiopathic progressive neuropathy |
| G608 | G60.8 Other hereditary and idiopathic neuropathies |
| G609 | G60.9 Hereditary and idiopathic neuropathy |
| G61 | G61 Inflammatory polyneuropathy |
| G610 | G61.0 Guillain-Barre syndrome |
| G611 | G61.1 Serum neuropathy |
| G618 | G61.8 Other inflammatory polyneuropathies |
| G619 | G61.9 Inflammatory polyneuropathy |
| G62 | G62 Other polyneuropathies |
| G620 | G62.0 Drug-induced polyneuropathy |
| G621 | G62.1 Alcoholic polyneuropathy |
| G622 | G62.2 Polyneuropathy due to other toxic agents |
| G628 | G62.8 Other specified polyneuropathies |
| G629 | G62.9 Polyneuropathy |
| G63 | G63 Polyneuropathy in diseases classified elsewhere |
| G630 | G63.0 Polyneuropathy in infectious and parasitic diseases classified elsewhere |
| G631 | G63.1 Polyneuropathy in neoplastic disease |
| G632 | G63.2 Diabetic polyneuropathy |
| G633 | G63.3 Polyneuropathy in other endocrine and metabolic diseases |
| G634 | G63.4 Polyneuropathy in nutritional deficiency |
| G635 | G63.5 Polyneuropathy in systemic connective tissue disorders |
| G636 | G63.6 Polyneuropathy in other musculoskeletal disorders |
| G638 | G63.8 Polyneuropathy in other diseases classified elsewhere |
| G64 | G64 Other disorders of peripheral nervous system |
| G70 | G70 Myasthenia gravis and other myoneural disorders |
| G700 | G70.0 Myasthenia gravis |
| G701 | G70.1 Toxic myoneural disorders |
| G702 | G70.2 Congenital and developmental myasthenia |
| G708 | G70.8 Other specified myoneural disorders |
| G709 | G70.9 Myoneural disorder |
| G71 | G71 Primary disorders of muscles |
| G710 | G71.0 Muscular dystrophy |
| G711 | G71.1 Myotonic disorders |
| G712 | G71.2 Congenital myopathies |
| G713 | G71.3 Mitochondrial myopathy |
| G718 | G71.8 Other primary disorders of muscles |
| G719 | G71.9 Primary disorder of muscle |
| G72 | G72 Other myopathies |
| G720 | G72.0 Drug-induced myopathy |
| G721 | G72.1 Alcoholic myopathy |
| G722 | G72.2 Myopathy due to other toxic agents |
| G723 | G72.3 Periodic paralysis |
| G724 | G72.4 Inflammatory myopathy |
| G728 | G72.8 Other specified myopathies |
| G729 | G72.9 Myopathy |
| G73 | G73 Disorders of myoneural junction and muscle in diseases classified elsewhere |
| G730 | G73.0 Myasthenic syndromes in endocrine diseases |
| G731 | G73.1 Eaton-Lambert syndrome |
| G732 | G73.2 Other myasthenic syndromes in neoplastic disease |
| G733 | G73.3 Myasthenic syndromes in other diseases classified elsewhere |
| G734 | G73.4 Myopathy in infectious and parasitic diseases classified elsewhere |
| G735 | G73.5 Myopathy in endocrine diseases |
| G736 | G73.6 Myopathy in metabolic diseases |
| G737 | G73.7 Myopathy other diseases classified elsewhere |
| G80 | G80 Infantile cerebral palsy |
| G800 | G80.0 Spastic cerebral palsy |
| G801 | G80.1 Spastic diplegia |
| G802 | G80.2 Infantile hemiplegia |
| G803 | G80.3 Dyskinetic cerebral palsy |
| G804 | G80.4 Ataxic cerebral palsy |
| G808 | G80.8 Other infantile cerebral palsy |
| G809 | G80.9 Infantile cerebral palsy |
| G81 | G81 Hemiplegia |
| G810 | G81.0 Flaccid hemiplegia |
| G811 | G81.1 Spastic hemiplegia |
| G819 | G81.9 Hemiplegia |
| G82 | G82 Paraplegia and tetraplegia |
| G820 | G82.0 Flaccid paraplegia |
| G821 | G82.1 Spastic paraplegia |
| G822 | G82.2 Paraplegia |
| G823 | G82.3 Flaccid tetraplegia |
| G824 | G82.4 Spastic tetraplegia |
| G825 | G82.5 Tetraplegia |
| G83 | G83 Other paralytic syndromes |
| G830 | G83.0 Diplegia of upper limbs |
| G831 | G83.1 Monoplegia of lower limb |
| G832 | G83.2 Monoplegia of upper limb |
| G833 | G83.3 Monoplegia |
| G834 | G83.4 Cauda equina syndrome |
| G835 | G83.5 Locked-in syndrome |
| G838 | G83.8 Other specified paralytic syndromes |
| G839 | G83.9 Paralytic syndrome |
| G90 | G90 Disorders of autonomic nervous system |
| G900 | G90.0 Idiopathic peripheral autonomic neuropathy |
| G901 | G90.1 Familial dysautonomia [Riley-Day] |
| G902 | G90.2 Horner's syndrome |
| G903 | G90.3 Multisystem degeneration |
| G904 | G90.4 Autonomic dysreflexia |
| G908 | G90.8 Other disorders of autonomic nervous system |
| G909 | G90.9 Disorder of autonomic nervous system |
| G91 | G91 Hydrocephalus |
| G910 | G91.0 Communicating hydrocephalus |
| G911 | G91.1 Obstructive hydrocephalus |
| G912 | G91.2 Normal-pressure hydrocephalus |
| G913 | G91.3 Posttraumatic hydrocephalus |
| G918 | G91.8 Other hydrocephalus |
| G919 | G91.9 Hydrocephalus |
| G92 | G92 Toxic encephalopathy |
| G93 | G93 Other disorders of brain |
| G930 | G93.0 Cerebral cysts |
| G931 | G93.1 Anoxic brain damage |
| G932 | G93.2 Benign intracranial hypertension |
| G933 | G93.3 Postviral fatigue syndrome |
| G934 | G93.4 Encephalopathy |
| G935 | G93.5 Compression of brain |
| G936 | G93.6 Cerebral oedema |
| G937 | G93.7 Reye's syndrome |
| G938 | G93.8 Other specified disorders of brain |
| G939 | G93.9 Disorder of brain |
| G94 | G94 Other disorders of brain in diseases classified elsewhere |
| G940 | G94.0 Hydrocephalus in infectious and parasitic diseases classified elsewhere |
| G941 | G94.1 Hydrocephalus in neoplastic disease |
| G942 | G94.2 Hydrocephalus in other diseases classified elsewhere |
| G948 | G94.8 Other specified disorders of brain in diseases classified elsewhere |
| G95 | G95 Other diseases of spinal cord |
| G950 | G95.0 Syringomyelia and syringobulbia |
| G951 | G95.1 Vascular myelopathies |
| G952 | G95.2 Cord compression |
| G958 | G95.8 Other specified diseases of spinal cord |
| G959 | G95.9 Disease of spinal cord |
| G96 | G96 Other disorders of central nervous system |
| G960 | G96.0 Cerebrospinal fluid leak |
| G961 | G96.1 Disorders of meninges |
| G968 | G96.8 Other specified disorders of central nervous system |
| G969 | G96.9 Disorder of central nervous system |
| G97 | G97 Postprocedural disorders of nervous system |
| G970 | G97.0 Cerebrospinal fluid leak from spinal puncture |
| G971 | G97.1 Other reaction to spinal and lumbar puncture |
| G972 | G97.2 Intracranial hypotension following ventricular shunting |
| G978 | G97.8 Other postprocedural disorders of nervous system |
| G979 | G97.9 Postprocedural disorder of nervous system |
| G98 | G98 Other disorders of nervous system |
| G99 | G99 Other disorders of nervous system in diseases classified elsewhere |
| G990 | G99.0 Autonomic neuropathy in endocrine and metabolic diseases |
| G991 | G99.1 Other disorders of autonomic nervous system in other diseases classified elsewhere |
| G992 | G99.2 Myelopathy in diseases classified elsewhere |
| G998 | G99.8 Other specified disorders of nervous system in diseases classified elsewhere |
| C70 | C70 Malignant neoplasm of meninges |
| C700 | C70.0 Cerebral meninges |
| C701 | C70.1 Spinal meninges |
| C709 | C70.9 Meninges, unspecified |
| C71 | C71 Malignant neoplasm of brain |
| C710 | C71.0 Cerebrum, except lobes and ventricles |
| C711 | C71.1 Frontal lobe |
| C712 | C71.2 Temporal lobe |
| C713 | C71.3 Parietal lobe |
| C714 | C71.4 Occipital lobe |
| C715 | C71.5 Cerebral ventricle |
| C716 | C71.6 Cerebellum |
| C717 | C71.7 Brain stem |
| C718 | C71.8 Overlapping lesion of brain |
| C719 | C71.9 Brain, unspecified |
| C72 | C72 Malignant neoplasm of spinal cord, cranial nerves and other parts of central nervous system |
| C720 | C72.0 Spinal cord |
| C721 | C72.1 Cauda equina |
| C722 | C72.2 Olfactory nerve |
| C723 | C72.3 Optic nerve |
| C724 | C72.4 Acoustic nerve |
| C725 | C72.5 Other and unspecified cranial nerves |
| C728 | C72.8 Overlapping lesion of brain and other parts of central nervous system |
| C729 | C72.9 Central nervous system, unspecified |
| D32 | D32 Benign neoplasm of meninges |
| D320 | D32.0 Cerebral meninges |
| D321 | D32.1 Spinal meninges |
| D329 | D32.9 Meninges, unspecified |
| D33 | D33 Benign neoplasm of brain and other parts of central nervous system |
| D330 | D33.0 Brain, supratentorial |
| D331 | D33.1 Brain, infratentorial |
| D332 | D33.2 Brain, unspecified |
| D333 | D33.3 Cranial nerves |
| D334 | D33.4 Spinal cord |
| D337 | D33.7 Other specified parts of central nervous system |
| D339 | D33.9 Central nervous system, unspecified |
| I60 | I60 Subarachnoid haemorrhage |
| I600 | I60.0 Subarachnoid haemorrhage from carotid siphon and bifurcation |
| I601 | I60.1 Subarachnoid haemorrhage from middle cerebral artery |
| I602 | I60.2 Subarachnoid haemorrhage from anterior communicating artery |
| I603 | I60.3 Subarachnoid haemorrhage from posterior communicating artery |
| I604 | I60.4 Subarachnoid haemorrhage from basilar artery |
| I605 | I60.5 Subarachnoid haemorrhage from vertebral artery |
| I606 | I60.6 Subarachnoid haemorrhage from other intracranial arteries |
| I607 | I60.7 Subarachnoid haemorrhage from intracranial artery, unspecified |
| I608 | I60.8 Other subarachnoid haemorrhage |
| I609 | I60.9 Subarachnoid haemorrhage, unspecified |
| I61 | I61 Intracerebral haemorrhage |
| I610 | I61.0 Intracerebral haemorrhage in hemisphere, subcortical |
| I611 | I61.1 Intracerebral haemorrhage in hemisphere, cortical |
| I612 | I61.2 Intracerebral haemorrhage in hemisphere, unspecified |
| I613 | I61.3 Intracerebral haemorrhage in brain stem |
| I614 | I61.4 Intracerebral haemorrhage in cerebellum |
| I615 | I61.5 Intracerebral haemorrhage, intraventricular |
| I616 | I61.6 Intracerebral haemorrhage, multiple localised |
| I618 | I61.8 Other intracerebral haemorrhage |
| I619 | I61.9 Intracerebral haemorrhage, unspecified |
| I62 | I62 Other nontraumatic intracranial haemorrhage |
| I620 | I62.0 Subdural haemorrhage (acute) (nontraumatic) |
| I621 | I62.1 Nontraumatic extradural haemorrhage |
| I629 | I62.9 Intracranial haemorrhage (nontraumatic), unspecified |
| I63 | I63 Cerebral infarction |
| I630 | I63.0 Cerebral infarction due to thrombosis of precerebral arteries |
| I631 | I63.1 Cerebral infarction due to embolism of precerebral arteries |
| I632 | I63.2 Cerebral infarction due to unspecified occlusion or stenosis of precerebral arteries |
| I633 | I63.3 Cerebral infarction due to thrombosis of cerebral arteries |
| I634 | I63.4 Cerebral infarction due to embolism of cerebral arteries |
| I635 | I63.5 Cerebral infarction due to unspecified occlusion or stenosis of cerebral arteries |
| I636 | I63.6 Cerebral infarction due to cerebral venous thrombosis, nonpyogenic |
| I638 | I63.8 Other cerebral infarction |
| I639 | I63.9 Cerebral infarction, unspecified |
| I64 | I64 Stroke, not specified as haemorrhage or infarction |
| I65 | I65 Occlusion and stenosis of precerebral arteries, not resulting in cerebral infarction |
| I650 | I65.0 Occlusion and stenosis of vertebral artery |
| I651 | I65.1 Occlusion and stenosis of basilar artery |
| I652 | I65.2 Occlusion and stenosis of carotid artery |
| I653 | I65.3 Occlusion and stenosis of multiple and bilateral precerebral arteries |
| I658 | I65.8 Occlusion and stenosis of other precerebral artery |
| I659 | I65.9 Occlusion and stenosis of unspecified precerebral artery |
| I66 | I66 Occlusion and stenosis of cerebral arteries, not resulting in cerebral infarction |
| I660 | I66.0 Occlusion and stenosis of middle cerebral artery |
| I661 | I66.1 Occlusion and stenosis of anterior cerebral artery |
| I662 | I66.2 Occlusion and stenosis of posterior cerebral artery |
| I663 | I66.3 Occlusion and stenosis of cerebellar arteries |
| I664 | I66.4 Occlusion and stenosis of multiple and bilateral cerebral arteries |
| I668 | I66.8 Occlusion and stenosis of other cerebral artery |
| I669 | I66.9 Occlusion and stenosis of unspecified cerebral artery |
| I67 | I67 Other cerebrovascular diseases |
| I670 | I67.0 Dissection of cerebral arteries, nonruptured |
| I671 | I67.1 Cerebral aneurysm, nonruptured |
| I672 | I67.2 Cerebral atherosclerosis |
| I673 | I67.3 Progressive vascular leukoencephalopathy |
| I674 | I67.4 Hypertensive encephalopathy |
| I675 | I67.5 Moyamoya disease |
| I676 | I67.6 Nonpyogenic thrombosis of intracranial venous system |
| I677 | I67.7 Cerebral arteritis, not elsewhere classified |
| I678 | I67.8 Other specified cerebrovascular diseases |
| I679 | I67.9 Cerebrovascular disease, unspecified |
| I68 | I68 Cerebrovascular disorders in diseases classified elsewhere |
| I680 | I68.0 Cerebral amyloid angiopathy |
| I681 | I68.1 Cerebral arteritis in infectious and parasitic diseases classified elsewhere |
| I682 | I68.2 Cerebral arteritis in other diseases classified elsewhere |
| I688 | I68.8 Other cerebrovascular disorders in diseases classified elsewhere |
| I69 | I69 Sequelae of cerebrovascular disease |
| I690 | I69.0 Sequelae of subarachnoid haemorrhage |
| I691 | I69.1 Sequelae of intracerebral haemorrhage |
| I692 | I69.2 Sequelae of other nontraumatic intracranial haemorrhage |
| I693 | I69.3 Sequelae of cerebral infarction |
| I694 | I69.4 Sequelae of stroke, not specified as haemorrhage or infarction |
| I698 | I69.8 Sequelae of other and unspecified cerebrovascular diseases |
| S000 | S00.0 Superficial injury of scalp |
| S001 | S00.1 Contusion of eyelid and periocular area |
| S002 | S00.2 Other superficial injuries of eyelid and periocular area |
| S003 | S00.3 Superficial injury of nose |
| S004 | S00.4 Superficial injury of ear |
| S005 | S00.5 Superficial injury of lip and oral cavity |
| S007 | S00.7 Multiple superficial injuries of head |
| S008 | S00.8 Superficial injury of other parts of head |
| S009 | S00.9 Superficial injury of head, part unspecified |
| S01 | S01 Open wound of head |
| S010 | S01.0 Open wound of scalp |
| S011 | S01.1 Open wound of eyelid and periocular area |
| S012 | S01.2 Open wound of nose |
| S013 | S01.3 Open wound of ear |
| S014 | S01.4 Open wound of cheek and temporomandibular area |
| S015 | S01.5 Open wound of lip and oral cavity |
| S017 | S01.7 Multiple open wounds of head |
| S018 | S01.8 Open wound of other parts of head |
| S019 | S01.9 Open wound of head, part unspecified |
| S02 | S02 Fracture of skull and facial bones |
| S020 | S02.0 Fracture of vault of skull |
| S0200 | S02.00 Fracture of vault of skull (closed) |
| S0201 | S02.01 Fracture of vault of skull (open) |
| S021 | S02.1 Fracture of base of skull |
| S0210 | S02.10 Fracture of base of skull (closed) |
| S0211 | S02.11 Fracture of base of skull (open) |
| S022 | S02.2 Fracture of nasal bones |
| S0220 | S02.20 Fracture of nasal bones (closed) |
| S0221 | S02.21 Fracture of nasal bones (open) |
| S023 | S02.3 Fracture of orbital floor |
| S0230 | S02.30 Fracture of orbital floor (closed) |
| S0231 | S02.31 Fracture of orbital floor (open) |
| S024 | S02.4 Fracture of malar and maxillary bones |
| S0240 | S02.40 Fracture of malar and maxillary bones (closed) |
| S0241 | S02.41 Fracture of malar and maxillary bones (open) |
| S025 | S02.5 Fracture of tooth |
| S0250 | S02.50 Fracture of tooth (closed) |
| S0251 | S02.51 Fracture of tooth (open) |
| S026 | S02.6 Fracture of mandible |
| S0260 | S02.60 Fracture of mandible (closed) |
| S0261 | S02.61 Fracture of mandible (open) |
| S027 | S02.7 Multiple fractures involving skull and facial bones |
| S0270 | S02.70 Multiple fractures involving skull and facial bones (closed) |
| S0271 | S02.71 Multiple fractures involving skull and facial bones (open) |
| S028 | S02.8 Fractures of other skull and facial bones |
| S0280 | S02.80 Fractures of other skull and facial bones (closed) |
| S0281 | S02.81 Fractures of other skull and facial bones (open) |
| S029 | S02.9 Fracture of skull and facial bones, part unspecified |
| S0290 | S02.90 Fracture of skull and facial bones, part unspecified (closed) |
| S0291 | S02.91 Fracture of skull and facial bones, part unspecified (open) |
| S03 | S03 Dislocation, sprain and strain of joints and ligaments of head |
| S030 | S03.0 Dislocation of jaw |
| S031 | S03.1 Dislocation of septal cartilage of nose |
| S032 | S03.2 Dislocation of tooth |
| S033 | S03.3 Dislocation of other and unspecified parts of head |
| S034 | S03.4 Sprain and strain of jaw |
| S035 | S03.5 Sprain and strain of joints and ligaments of other and unspecified parts of head |
| S04 | S04 Injury of cranial nerves |
| S040 | S04.0 Injury of optic nerve and pathways |
| S041 | S04.1 Injury of oculomotor nerve |
| S042 | S04.2 Injury of trochlear nerve |
| S043 | S04.3 Injury of trigeminal nerve |
| S044 | S04.4 Injury of abducent nerve |
| S045 | S04.5 Injury of facial nerve |
| S046 | S04.6 Injury of acoustic nerve |
| S047 | S04.7 Injury of accessory nerve |
| S048 | S04.8 Injury of other cranial nerves |
| S049 | S04.9 Injury of unspecified cranial nerve |
| S05 | S05 Injury of eye and orbit |
| S050 | S05.0 Injury of conjunctiva and corneal abrasion without mention of foreign body |
| S051 | S05.1 Contusion of eyeball and orbital tissues |
| S052 | S05.2 Ocular laceration and rupture with prolapse or loss of intraocular tissue |
| S053 | S05.3 Ocular laceration without prolapse or loss of intraocular tissue |
| S054 | S05.4 Penetrating wound of orbit with or without foreign body |
| S055 | S05.5 Penetrating wound of eyeball with foreign body |
| S056 | S05.6 Penetrating wound of eyeball without foreign body |
| S057 | S05.7 Avulsion of eye |
| S058 | S05.8 Other injuries of eye and orbit |
| S059 | S05.9 Injury of eye and orbit, part unspecified |
| S06 | S06 Intracranial injury |
| S060 | S06.0 Concussion |
| S0600 | S06.00 Concussion (without open intracranial wound) |
| S0601 | S06.01 Concussion (with open intracranial wound) |
| S061 | S06.1 Traumatic cerebral oedema |
| S0610 | S06.10 Traumatic cerebral oedema (without open intracranial wound) |
| S0611 | S06.11 Traumatic cerebral oedema (with open intracranial wound) |
| S062 | S06.2 Diffuse brain injury |
| S0620 | S06.20 Diffuse brain injury (without open intracranial wound) |
| S0621 | S06.21 Diffuse brain injury (with open intracranial wound) |
| S063 | S06.3 Focal brain injury |
| S0630 | S06.30 Focal brain injury (without open intracranial wound) |
| S0631 | S06.31 Focal brain injury (with open intracranial wound) |
| S064 | S06.4 Epidural haemorrhage |
| S0640 | S06.40 Epidural haemorrhage (without open intracranial wound) |
| S0641 | S06.41 Epidural haemorrhage (with open intracranial wound) |
| S065 | S06.5 Traumatic subdural haemorrhage |
| S0650 | S06.50 Traumatic subdural haemorrhage (without open intracranial wound) |
| S0651 | S06.51 Traumatic subdural haemorrhage (with open intracranial wound) |
| S066 | S06.6 Traumatic subarachnoid haemorrhage |
| S0660 | S06.60 Traumatic subarachnoid haemorrhage (without open intracranial wound) |
| S0661 | S06.61 Traumatic subarachnoid haemorrhage (with open intracranial wound) |
| S067 | S06.7 Intracranial injury with prolonged coma |
| S0670 | S06.70 Intracranial injury with prolonged coma (without open intracranial wound) |
| S0671 | S06.71 Intracranial injury with prolonged coma (with open intracranial wound) |
| S068 | S06.8 Other intracranial injuries |
| S0680 | S06.80 Other intracranial injuries (without open intracranial wound) |
| S0681 | S06.81 Other intracranial injuries (with open intracranial wound) |
| S069 | S06.9 Intracranial injury, unspecified |
| S0690 | S06.90 Intracranial injury, unspecified (without open intracranial wound) |
| S0691 | S06.91 Intracranial injury, unspecified (with open intracranial wound) |
| S07 | S07 Crushing injury of head |
| S070 | S07.0 Crushing injury of face |
| S071 | S07.1 Crushing injury of skull |
| S078 | S07.8 Crushing injury of other parts of head |
| S079 | S07.9 Crushing injury of head, part unspecified |
| S08 | S08 Traumatic amputation of part of head |
| S080 | S08.0 Avulsion of scalp |
| S081 | S08.1 Traumatic amputation of ear |
| S088 | S08.8 Traumatic amputation of other parts of head |
| S089 | S08.9 Traumatic amputation of unspecified part of head |
| S09 | S09 Other and unspecified injuries of head |
| S090 | S09.0 Injury of blood vessels of head, not elsewhere classified |
| S091 | S09.1 Injury of muscle and tendon of head |
| S092 | S09.2 Traumatic rupture of ear drum |
| S097 | S09.7 Multiple injuries of head |
| S098 | S09.8 Other specified injuries of head |
| S099 | S09.9 Unspecified injury of head |

*Table S3: List of 101 regions extracted from FreeSurfer using the Desikan-Killiany and ASEG atlases (Desikan et al., 2006; Fischl et al., 2002).*

'Left-Lateral-Ventricle',
'Left-Inf-Lat-Vent',
‘Left-Cerebellum-White-Matter',
‘Left-Cerebellum-Cortex',
‘Left-Thalamus-Proper',
‘Left-Caudate',
‘Left-Putamen',
‘Left-Pallidum',
‘3rd-Ventricle',
‘4th-Ventricle',
‘Brain-Stem',
‘Left-Hippocampus',
‘Left-Amygdala',
‘CSF',
‘Left-Accumbens-area',
‘Left-VentralDC',
‘Right-Lateral-Ventricle',
‘Right-Inf-Lat-Vent',
‘Right-Cerebellum-White-Matter',
‘Right-Cerebellum-Cortex',
‘Right-Thalamus-Proper',
‘Right-Caudate',
‘Right-Putamen',
‘Right-Pallidum',
‘Right-Hippocampus',
‘Right-Amygdala',
‘Right-Accumbens-area',
‘Right-VentralDC',
‘CC_Posterior',
‘CC_Mid_Posterior',
‘CC_Central',
‘CC_Mid_Anterior',
‘CC_Anterior',
‘lh_bankssts_volume',
‘lh_caudalanteriorcingulate_volume',
‘lh_caudalmiddlefrontal_volume',
‘lh_cuneus_volume',
‘lh_entorhinal_volume',
‘lh_fusiform_volume',
‘lh_inferiorparietal_volume',
‘lh_inferiortemporal_volume',
‘lh_isthmuscingulate_volume',
‘lh_lateraloccipital_volume',
‘lh_lateralorbitofrontal_volume',
‘lh_lingual_volume',
‘lh_medialorbitofrontal_volume',
‘lh_middletemporal_volume',
‘lh_parahippocampal_volume',
‘lh_paracentral_volume',
‘lh_parsopercularis_volume',
'lh_parsorbitalis_volume',
'lh_parstriangularis_volume',
'lh_pericalcarine_volume',
'lh_postcentral_volume',
'lh_posteriorcingulate_volume',
'lh_precentral_volume',
'lh_precuneus_volume',
'lh_rostralanteriorcingulate_volume',
'lh_rostralmiddlefrontal_volume',
'lh_superiorfrontal_volume',
'lh_superiorparietal_volume',
'lh_superiortemporal_volume',
'lh_supramarginal_volume',
'lh_frontalpole_volume',
'lh_temporalpole_volume',
'lh_transversetemporal_volume',
'lh_insula_volume',
'rh_bankssts_volume',
'rh_caudalanteriorcingulate_volume',
'rh_caudalmiddlefrontal_volume',
'rh_cuneus_volume',
'rh_entorhinal_volume',
'rh_fusiform_volume',
'rh_inferiorparietal_volume',
'rh_inferiortemporal_volume',
'rh_isthmuscingulate_volume',
rh_lateraloccipital_volume',
'rh_lateralorbitofrontal_volume',
'rh_lingual_volume',
'rh_medialorbitofrontal_volume',
'rh_middletemporal_volume',
'rh_parahippocampal_volume',
'rh_paracentral_volume',
'rh_parsopercularis_volume',
'rh_parsorbitalis_volume',
'rh_parstriangularis_volume',
'rh_pericalcarine_volume',
'rh_postcentral_volume',
'rh_posteriorcingulate_volume',
'rh_precentral_volume',
'rh_precuneus_volume',
'rh_rostralanteriorcingulate_volume',
'rh_rostralmiddlefrontal_volume',
'rh_superiorfrontal_volume',
'rh_superiorparietal_volume',
'rh_superiortemporal_volume',
'rh_supramarginal_volume',
'rh_frontalpole_volume',
'rh_temporalpole_volume',
'rh_transversetemporal_volume',
'rh_insula_volume'

*Figure S1: Age and sex distribution in UK Biobank dataset from sites 1 and 2.*

*Abbreviations: F, female; M, male*


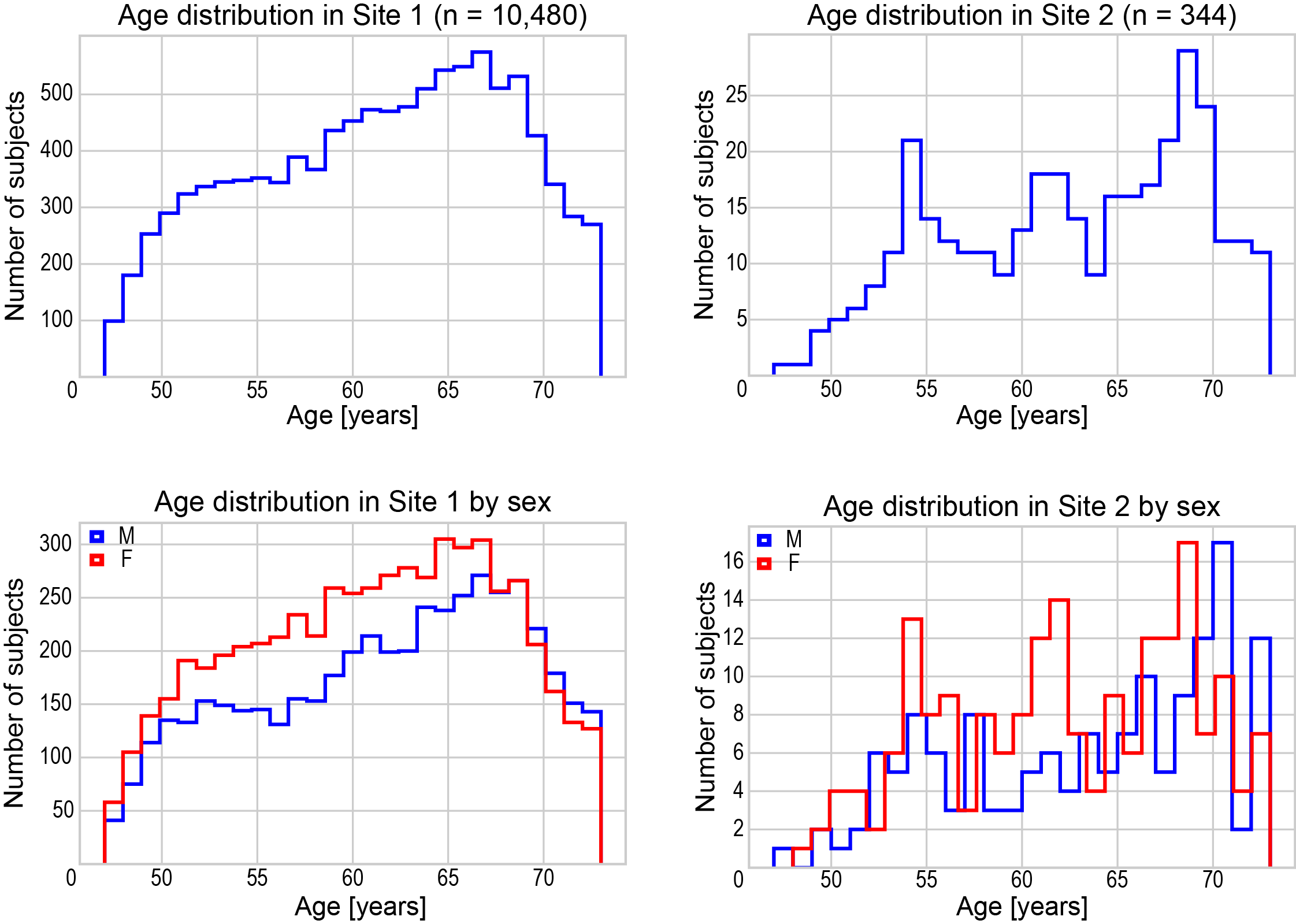


**References for supplementary materials**

Ashburner, J. (2007). A fast diffeomorphic image registration algorithm. *NeuroImage*, *38*, 95–113. https://doi.org/10.1016/j.neuroimage.2007.07.007

Aycheh, H. M., Seong, J. K., Shin, J. H., Na, D. L., Kang, B., Seo, S. W., & Sohn, K.-A. (2018). Biological brain age prediction using cortical thickness data: A large scale cohort study. *Frontiers in Aging Neuroscience*, *10*(252), 1–14. https://doi.org/10.3389/fnagi.2018.00252

Cole, J. H., Leech, R., & Sharp, D. J. (2015). Prediction of brain age suggests accelerated atrophy after traumatic brain injury. *Annals of Neurology*, *77*(4), 571–581. https://doi.org/10.1002/ana.24367

Cole, J. H., Ritchie, S. J., Bastin, M. E., Valdés Hernández, M. C., Muñoz Maniega, S., Royle, N., Corley, J., Pattie, A., Harris, S. E., Zhang, Q., Wray, N. R., Redmond, P., Marioni, R. E., Starr, J. M., Cox, S. R., Wardlaw, J. M., Sharp, D. J., & Deary, I. J. (2018). Brain age predicts mortality. *Molecular Psychiatry*, *23*, 1385–1392. https://doi.org/10.1038/mp.2017.62

Desikan, R. S., Ségonne, F., Fischl, B., Quinn, B. T., Dickerson, B. C., Blacker, D., Buckner, R. L., Dale, A. M., Maguire, R. P., Hyman, B. T., Albert, M. S., & Killiany, R. J. (2006). An automated labeling system for subdividing the human cerebral cortex on MRI scans into gyral based regions of interest. *NeuroImage*, *31*, 968–980. https://doi.org/10.1016/j.neuroimage.2006.01.021

Fischl, B., Salat, D. H., Busa, E., Albert, M., Dieterich, M., Haselgrove, C., van der Kouwe, A., Killiany, R., Kennedy, D., Klaveness, S., Montillo, A., Makris, N., Rosen, B., & Dale, A. M. (2002). Whole brain segmentation: Automated labeling of neuroanatomical structures in the human brain. *Neuron*, *33*, 341–355. https://surfer.nmr.mgh.harvard.edu/ftp/articles/fischl02-labeling.pdf

Franke, K., Ziegler, G., Klöppel, S., & Gaser, C. (2010). Estimating the age of healthy subjects from T1-weighted MRI scans using kernel methods: Exploring the influence of various parameters. *NeuroImage*, *50*(3), 883–892. https://doi.org/10.1016/j.neuroimage.2010.01.005

Fujimoto, R., Kondo, C., Ito, K., Wu, K., Sato, K., Taki, Y., Fukuda, H., & Aoki, T. (2016). Age estimation using effective brain local features from T1-weighted images. *Proceedings of the Annual International Conference of the IEEE Engineering in Medicine and Biology Society, EMBS*, *October*, 5941–5944. https://doi.org/10.1109/EMBC.2016.7592081

Gutierrez Becker, B., Klein, T., & Wachinger, C. (2018). Gaussian process uncertainty in age estimation as a measure of brain abnormality. *NeuroImage*, *175*(March), 246–258. https://doi.org/10.1016/j.neuroimage.2018.03.075

Kondo, C., Ito, K., Wu, K., Sato, K., Taki, Y., Fukuda, H., & Aoki, T. (2015). An age estimation method using brain local features for T1-weighted images. *Proceedings of the Annual International Conference of the IEEE Engineering in Medicine and Biology Society, EMBS*, *November*, 666–669. https://doi.org/10.1109/EMBC.2015.7318450

Koutsouleris, N., Davatzikos, C., Borgwardt, S., Gaser, C., Bottlender, R., Frodl, T., Falkai, P., Riecher-Rössler, A., Möller, H. J., Reiser, M., Pantelis, C., & Meisenzahl, E. (2014). Accelerated brain aging in schizophrenia and beyond: A neuroanatomical marker of psychiatric disorders. *Schizophrenia Bulletin*, *40*(5), 1140–1153. https://doi.org/10.1093/schbul/sbt142

Lancaster, J., Lorenz, R., Leech, R., & Cole, J. H. (2018). Bayesian optimization for neuroimaging pre-processing in brain age classification and prediction. *Frontiers in Aging Neuroscience*, *10*(28). https://doi.org/10.3389/fnagi.2018.00028

Le, T. T., Kuplicki, R. T., McKinney, B. A., Yeh, H.-W., Thompson, W. K., Paulus, M. P., & Tulsa 1000 Investigators. (2018). A nonlinear simulation framework supports adjusting for age when analyzing BrainAGE. *Frontiers in Aging Neuroscience*, *10*(317), 1–11. https://doi.org/10.3389/fnagi.2018.00317

Liem, F., Varoquaux, G., Kynast, J., Beyer, F., Kharabian Masouleh, S., Huntenburg, J. M., Lampe, L., Rahim, M., Abraham, A., Craddock, R. C., Riedel-Heller, S., Luck, T., Loeffler, M., Schroeter, M. L., Witte, A. V., Villringer, A., & Margulies, D. S. (2017). Predicting brain-age from multimodal imaging data captures cognitive impairment. *NeuroImage*, *148*(July 2016), 179–188. https://doi.org/10.1016/j.neuroimage.2016.11.005

Madan, C. R., & Kensinger, E. A. (2018). Predicting age from cortical structure across the lifespan. *European Journal of Neuroscience*, *47*, 399–416. https://doi.org/10.1111/ejn.13835

Su, L., Wang, L., & Hu, D. (2013). Predicting the age of healthy adults from structural MRI by sparse representation. In J. Yang, F. Fang, & C. Sun (Eds.), *Intelligent Science and Intelligent Data Engineering IScIDE 2012* (pp. 271–279). Springer. https://doi.org/10.1007/978-3-642-36669-7_5

Valizadeh, S. A., Hänggi, J., Mérillat, S., & Jäncke, L. (2017). Age prediction on the basis of brain anatomical measures. *Human Brain Mapping*, *38*(2), 997–1008. https://doi.org/10.1002/hbm.23434

Wang, J., Li, W., Miao, W., Dai, D., Hua, J., & He, H. (2014). Age estimation using cortical surface pattern combining thickness with curvatures. *Medical and Biological Engineering and Computing*, *52*, 331–341. https://doi.org/10.1007/s11517-013-1131-9
